# Acalabrutinib vs chlorambucil plus rituximab in untreated chronic lymphocytic leukemia: A randomized phase 3 Asian study

**DOI:** 10.1007/s00277-026-07006-z

**Published:** 2026-05-19

**Authors:** Jia Li, Shuhua Yi, Thanh Nguyen, Wenjuan Yu, Keshu Zhou, Haiwen Huang, Udomsak Bunworasate, Man Huynh, Hui Zhou, Su-Peng Yeh, Yiqiu Wang, Xiaofeng Liu, Wei Fu, Lugui Qiu

**Affiliations:** 1 Department of Hematology, Shengjing Hospital of China Medical University, Shenyang, China; 2 State Key Laboratory of Experimental Hematology, National Clinical Research Center for Blood Diseases, Haihe Laboratory of Cell Ecosystem, Institute of Hematology & Blood Diseases Hospital, Chinese Academy of Medical Sciences & Peking Union Medical College, 288 Nanjing Road, Tianjin, 300020 China; 3 Tianjin Institutes of Health Science, Tianjin, China; 4 National Institute of Hematology and Blood Transfusion, Hanoi, Vietnam; 5 Department of Hematology, The First Affiliated Hospital, Zhejiang University College of Medicine, Hangzhou, China; 6 Department of Hematology, The Affiliated Cancer Hospital of Zhengzhou University, Henan Cancer Hospital, Zhengzhou, China; 7 The First Hospital of Soochow University, Suzhou, China; 8 Department of Medicine, Faculty of Medicine, Chulalongkorn University, Bangkok, Thailand; 9 Blood Transfusion Hematology Hospital, Ho Chi Minh City, Vietnam; 10 Department of Lymphoma & Hematology, The Affiliated Cancer Hospital of Xiangya School of Medicine, Hunan Cancer Hospital, Central South University, Changsha, China; 11 Division of Hematology and Oncology, Department of Internal Medicine, China Medical University Hospital, Taichung, Taiwan; 12 AstraZeneca, Shanghai, China

**Keywords:** Bruton tyrosine kinase inhibitor, Chemoimmunotherapy, Chronic lymphocytic leukemia, Phase 3, Clinical Trial, Asia

## Abstract

Acalabrutinib is a selective, covalent Bruton tyrosine kinase inhibitor approved in multiple countries for patients with chronic lymphocytic leukemia (CLL). Acalabrutinib is approved in China for patients with CLL who received ≥ 1 prior therapy. We report results from a randomized, multicenter, open-label, phase 3 study (NCT04075292) evaluating the efficacy and safety of acalabrutinib vs chlorambucil plus rituximab (C + R) in medically unfit patients with treatment-naive CLL in Asia. As of January 3, 2024, 155 patients (overall cohort: acalabrutinib, *n* = 77; C + R, *n* = 78; China cohort [mainland China and Taiwan], *n* = 53 and *n* = 50, respectively) were enrolled. With a median follow-up of 23.5 and 18.2 months in the overall and China cohorts, respectively, acalabrutinib demonstrated a 92% risk reduction of disease progression or death vs C + R (both cohorts: hazard ratio, 0.08; *P* < 0.0001; primary endpoint). Overall response rates were 76.6% vs 71.8% with median duration of response not reached vs 11.6 months in the acalabrutinib vs C + R arms, respectively. In the acalabrutinib arm, no atrial fibrillation or hypertension events were observed. Efficacy and safety were consistent across cohorts. Acalabrutinib was well tolerated and demonstrated a progression-free survival benefit vs C + R in this Asian population.

**Supplementary Information:**

The online version contains supplementary material available at 10.1007/s00277-026-07006-z.

## Introduction

Chronic lymphocytic leukemia (CLL) is an incurable disease that occurs primarily in older patients [1, 2]. CLL has a variable disease course; many patients do not require treatment for years and have survival equal to that of age-matched controls, but other patients have aggressive disease and poor prognoses [2].

Historical first-line therapy comprising chemoimmunotherapy (CIT) and anti-CD20 antibodies greatly improved outcomes [3–5]. However, CIT and anti-CD20 antibodies have largely been replaced by targeted therapies including Bruton tyrosine kinase inhibitors (BTKis) and B-cell lymphoma 2 inhibitors (BCL-2is) with or without anti-CD20 antibodies [6–8]. Acalabrutinib is a highly selective, covalent BTKi approved for use in combination with bendamustine and rituximab for adults with previously untreated mantle cell lymphoma who are ineligible for autologous stem cell transplant and as monotherapy for the treatment of adults with relapsed or refractory (R/R) mantle cell lymphoma in the United States and in Europe; and for the treatment of CLL or small lymphocytic lymphoma (SLL) in the United States and other countries [9, 10]. The global, phase 3 ELEVATE-TN study of patients with treatment-naive (TN) CLL demonstrated that acalabrutinib with or without obinutuzumab yielded superior progression-free survival (PFS) with a manageable safety profile compared with obinutuzumab plus chlorambucil [11, 12]. Acalabrutinib plus obinutuzumab additionally demonstrated significant overall survival (OS) benefit compared with obinutuzumab plus chlorambucil at a median follow-up of 74.5 months [12]. Acalabrutinib has recently been approved in China for the treatment of patients with CLL who have received ≥ 1 prior therapy based on both the phase 3 ASCEND trial of acalabrutinib monotherapy vs investigator’s choice of idelalisib plus rituximab or bendamustine plus rituximab in patients with R/R CLL and a phase 1/2 trial of acalabrutinib in Chinese patients with R/R CLL [13–15].

Here we report results from a phase 3 study that evaluated the efficacy and safety of acalabrutinib vs chlorambucil in combination with rituximab (C + R) in unfit patients with TN CLL in sites across Asia. C + R was selected as the comparator in this trial based on its classification as a preferred regimen per the 2018 Chinese CLL/SLL Diagnostic and Treatment Guideline for medically unfit patients with CLL [16].

## Materials/patients and methods

### Study population

ChangE (NCT04075292) was a randomized, regional, multicenter, open-label phase 3 study that enrolled patients in mainland China, the Philippines, Taiwan, Thailand, and Vietnam. Eligible patients had TN CLL requiring treatment per International Workshop on CLL (iwCLL) 2018 criteria [17] and an Eastern Cooperative Oncology Group performance status (ECOG PS) ≤ 2. Patients were aged ≥ 65 years or aged > 18 and < 65 years with ≥ 1 of the following: creatinine clearance 30–69 mL/min or Cumulative Illness Rating Scale–Geriatric score > 6. Exclusion criteria included del(17p) or *TP53* mutations, significant cardiovascular disease (eg, uncontrolled or untreated symptomatic arrhythmias, congestive heart failure, or myocardial infarction within 6 months of screening, or any class 3 or 4 cardiac disease as defined by the New York Heart Association Functional Classification at screening), transformation of CLL to aggressive non-Hodgkin lymphoma, or central nervous system involvement by leukemia. See Online Resource 1 for complete inclusion/exclusion criteria.

This study was conducted in accordance with the consensus ethical principles derived from international guidelines including the Declaration of Helsinki. Independent ethics review committees within each institution reviewed and approved the study protocol. All patients provided written informed consent.

### Randomization and procedures

Patients were randomized 1:1 to receive acalabrutinib 100 mg orally twice daily continuously or combination C + R for a maximum of six 28-day cycles (chlorambucil; 0.5 mg/kg orally on days 1 and 15 of cycle [C] 1–6; rituximab, 375 mg/m^2^ intravenously on day 1 of C1 and 500 mg/m^2^ on day 1 of C2–6). Randomization was stratified by ECOG PS (0–1 vs 2) and Rai stage (0–II vs III–IV). Patients in the acalabrutinib arm were treated until disease progression or unacceptable toxicity. All patients were followed until death, withdrawal of consent, loss to follow-up, or study termination. Patients in the C + R arm were allowed to cross over to acalabrutinib after blinded independent central review (BICR)-confirmed progression.

### Endpoints and outcomes

The primary endpoint was BICR-assessed PFS according to iwCLL 2018 criteria [17]. Key secondary endpoints included BICR- and investigator-assessed overall response rate (ORR) and duration of response (DoR) according to iwCLL 2018 criteria [17], time to next treatment (TTNT), OS, and safety and tolerability evaluated in terms of adverse events (AEs), vital signs, clinical laboratory tests, physical examinations, and electrocardiograms. AEs were coded using the Medical Dictionary for Regulatory Activities version 26.1 and classified according to the Common Terminology Criteria for Adverse Events version 5.0. See Online Resource 1 for additional details.

### Statistical analysis

A sample size of 150 patients (75 patients randomized to each arm) was selected to achieve more than 95% power to detect a hazard ratio of 0.333 in PFS at the 2-sided significance level of 0.05. Descriptive statistics were used for all variables and are presented by treatment group. Unless otherwise stated, continuous variables were summarized by the number of observations, mean, standard deviation, median, minimum, and maximum; categorical variables were summarized by frequency counts and percentages for each category. Unless otherwise stated, percentages were calculated out of the population total for the corresponding treatment arm.

In general, the baseline value for statistical analysis was defined as the last non-missing value prior to administration of the first dose of study drug, except for efficacy variables. For efficacy variables, baseline was defined as the last assessment prior to randomization.

Safety data were summarized for the safety analysis set. Efficacy analyses were performed in the intention-to-treat population (all randomized patients) and analyzed according to randomized treatment arm. The primary endpoint tested the null hypothesis that BICR-assessed PFS in the acalabrutinib arm and C + R arm are the same. PFS was defined as the time from date of randomization until disease progression (assessed by the BICR per iwCLL 2018 criteria [17]) or death from any cause, whichever occurred first. The primary analysis compared PFS as assessed by BICR using a 2-sided stratified log-rank test, adjusting for ECOG PS (0–1 vs 2) and Rai stage (0–II vs III–IV) at randomization. The estimate of the hazard ratio (HR) and its corresponding 95% confidence interval (CI) was computed using a Cox proportional-hazards model stratified by the randomization strata. The distribution of PFS was summarized in each treatment arm using median and corresponding 95% CI based on Kaplan–Meier estimates. Subgroup analyses comparing PFS between the acalabrutinib arm and the C + R arm were conducted in various patient subgroups. See Online Resource 1 for additional details.

## Results

**Fig. 1:**
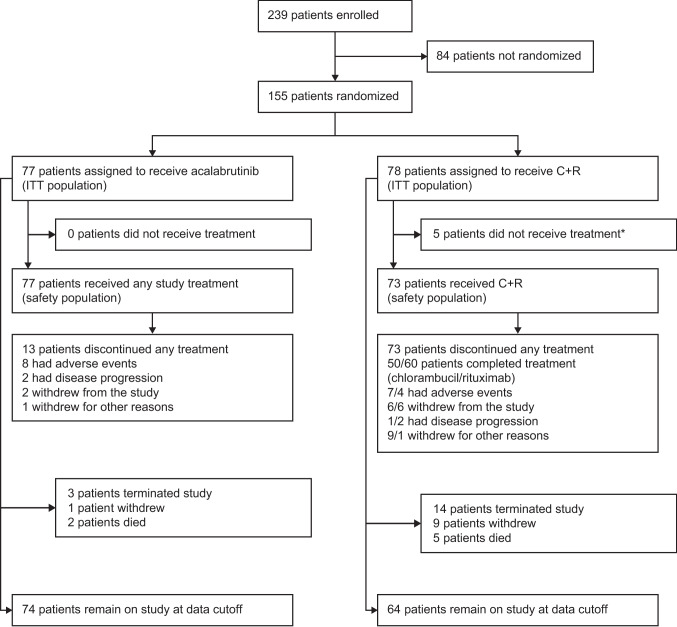
Patient disposition. *Four patients withdrew consent shortly after learning they were randomized to C + R. One patient with *TP53* mutation was incorrectly randomized to C + R and should have been excluded from the study; the patient withdrew consent before receiving any study treatment. *C* + *R* chlorambucil plus rituximab; *ITT* intention-to-treat

Between January 20, 2020, and February 17, 2023, 155 patients (acalabrutinib, *n* = 77; C + R, *n* = 78) enrolled across all 44 sites in Asia were included in the overall cohort. Of the 155 patients, 103 (acalabrutinib, *n* = 53; C + R, *n* = 50) from 30 sites across mainland China and Taiwan were included in the China cohort. Demographics and baseline characteristics were comparable across the 2 treatment arms in the overall and China cohorts (Table 1**; **Online Resource 1 Table S1). Patient disposition for the overall cohort is shown in Fig. 1; at the study cutoff of January 3, 2024, and at a median follow-up of 24 months in the overall cohort, 74 (96.1%) patients in the acalabrutinib arm and 64 (83.1%) in the C + R arm remained on study. Treatment was ongoing in 64 (42.7%) patients, and 86 (57.3%) had discontinued treatment. In the acalabrutinib arm, all 77 randomized patients received treatment; 13 (16.9%) patients discontinued acalabrutinib, including discontinuation due to AEs (*n* = 8, 10.4%), disease progression (*n* = 2, 2.6%), withdrawal by patient (*n* = 2, 2.6%), and diagnosis of atypical mantle cell lymphoma (*n* = 1, 1.3%). In the C + R arm, 73 (93.6%) of 78 randomized patients received treatment, among whom 50 (68.5%) completed chlorambucil treatment and 60 (82.2%) completed rituximab treatment; reasons for treatment withdrawal included AEs (chlorambucil, *n* = 7 [9.6%]; rituximab, *n* = 4 [5.5%]) and disease progression (chlorambucil, *n* = 1 [1.4%]; rituximab, *n* = 2 [2.7%]). A total of 24 (30.8%) patients in the C + R arm crossed over to the acalabrutinib arm. A total of 27 (35.1%) patients in the acalabrutinib arm and 17 (21.8%) patients in the C + R arm had ≥ 1 study disruption due to the COVID-19 pandemic, including study visit impacted (*n* = 26 and *n* = 17, respectively) and study drug impacted (*n* = 9 and *n* = 4, respectively); no patient withdrew from the study due to the COVID-19 pandemic (Online Resource 1 Table S2).

**Table 1:** Patient demographics and baseline characteristics

	Overall cohort		China cohort	
Characteristic	Acalabrutini (*n* = 77)	C + R (*n* = 78)	Acala (*n* = 53)	C + R (*n* = 50)
Age, median (range), y	64.0 (38–86)	67.0 (21–87)	64.0 (43–79)	65.0 (21–86)
Male sex, *n* (%)	43 (55.8)	47 (60.3)	30 (56.6)	32 (64.0)
ECOG PS, *n* (%)				
0–1	72 (93.5)	71 (91.0)	51 (96.2)	46 (92.0)
2	5 (6.5)	7 (9.0)	2 (3.8)	4 (8.0)
Unfit criteria, *n* (%)				
Age ≥ 65 y	35 (45.5)	46 (59.0)	25 (47.2)	28 (56.0)
Age > 18 to < 65 y	42 (54.5)	32 (41.0)	28 (52.8)	22 (44.0)
Creatinine clearance 30–69 mL/min	13 (16.9)	11 (14.1)	8 (15.1)	4 (8.0)
CIRS-G > 6	32 (41.6)	27 (34.6)	23 (43.4)	20 (40.0)
Both of the above	5 (6.5)	6 (7.7)	3 (5.7)	2 (4.0)
None of the above	2 (2.6)^a^	0	0	0
Rai stage III or IV, *n* (%)	44 (57.1)	44 (56.4)	31 (58.5)	27 (54.0)
Cytogenetic abnormality, *n* (%)				
del(11q)	15 (19.5)	10 (12.8)	12 (22.6)	8 (16.0)
Complex karyotype^b^	13 (16.9)	15 (19.2)	10 (18.9)	11 (22.0)
IGHV unmutated	32 (41.6)	30 (38.5)	22 (41.5)	19 (38.0)

^a^Two patients in the overall acalabrutinib arm had creatinine clearance < 69 mL/min during the screening period, which met the inclusion criteria; their creatinine clearances improved spontaneously and transiently on cycle 1 day 1

^b^Defined as patients with ≥ 3 abnormalities with ≥ 1 structural abnormality excluding inversion of chromosome 9

*C* + *R* chlorambucil plus rituximab; *CIRS-G* Cumulative Illness Rating Scale–Geriatric; *ECOG PS* Eastern Cooperative Oncology Group performance status; *IGHV* immunoglobulin heavy-chain variable region genes

### Efficacy

**Fig. 2:**
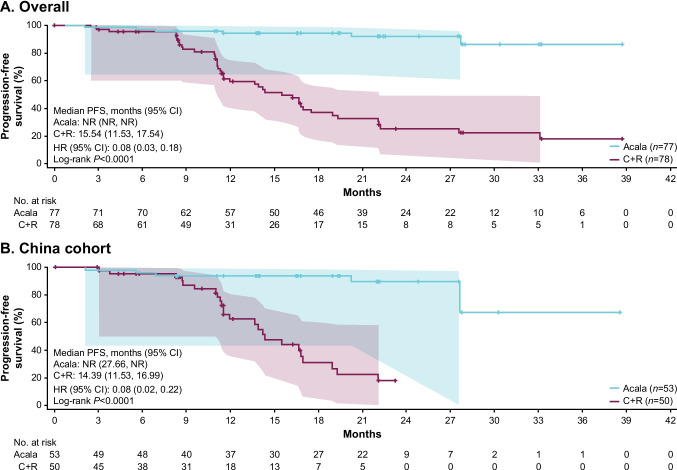
BICR-assessed PFS in (**A**) the overall cohort and (**B**) the China cohort. Median PFS was calculated using the Kaplan–Meier technique. CI for median PFS was derived based on the Brookmeyer–Crowley method. The analysis was performed using the unstratified log-rank test. The *P*-value is nominal for the China cohort. The HR was calculated using the unstratified Cox model with treatment as the only covariate. The CI for the HR was calculated using the profile likelihood. HR < 1 favors acalabrutinib. *Acala* acalabrutinib; *BICR* blinded independent central review; *CI* confidence interval; *C* + *R* chlorambucil plus rituximab; *HR* hazard ratio; *NR* not reached; *PFS* progression-free survival

At a median study follow-up of 23.5 (range, 0.0–44.4) months in the overall cohort, the study met its primary endpoint of BICR-assessed PFS; acalabrutinib demonstrated a 92% risk reduction of BICR-assessed disease progression or death vs C + R (HR, 0.08 [95% CI, 0.03, 0.18]; *P* < 0.0001; Fig. 2A). Median PFS was not reached (NR; 95% CI, not estimable [NE], NE) in the acalabrutinib arm vs 15.5 months (95% CI, 11.53, 17.54) in the C + R arm, with estimated 18-month PFS rates of 94% (95% CI, 85, 98) and 37% (95% CI, 24, 50), respectively, and estimated 24-month PFS rates of 92% (95% CI, 81, 97) and 25% (95% CI, 14, 38), respectively. Similar findings were reported for investigator-assessed PFS, with an 88% risk reduction of disease progression or death with acalabrutinib vs C + R (HR, 0.12 [95% CI, 0.05, 0.27]; *P* < 0.0001; Online Resource 1 Fig. S1). In subgroup analyses of BICR-assessed PFS divided by age, sex, ECOG PS, immunoglobulin heavy-chain variable region genes (IGHV) mutation status, complex karyotype status, country, Rai stage, β2-microglobulin level, bulky disease status, and presence of 11q deletion, HRs similarly demonstrated risk reduction of disease progression or death with acalabrutinib vs C + R for all subgroups (Online Resource 1 Fig. S2). Kaplan–Meier curves for BICR-assessed PFS by 11q deletion status and by IGHV mutation status are shown in Online Resource 1 Fig. S3 and S4.

**Fig. 3:**
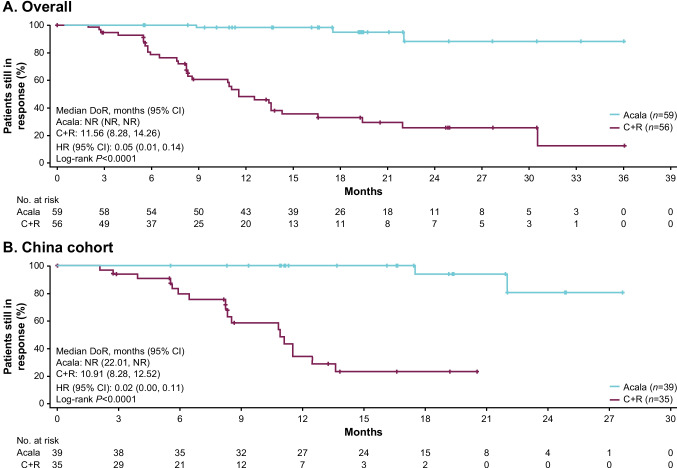
BICR-assessed duration of response in (**A**) the overall cohort and (**B**) the China cohort. The analysis included patients who had a response (defined as CR, CRi, nPR, or PR). Median DoR was calculated using the Kaplan–Meier method. CI for median DoR was derived based on the Brookmeyer–Crowley method. The analysis was performed using the unstratified log-rank test. The *P*-values are nominal for both cohorts. The HR was calculated using the unstratified Cox model with treatment as the only covariate. The CI for the HR was calculated using the profile likelihood. HR < 1 favors acalabrutinib. *Acala* acalabrutinib; *BICR* blinded independent central review; *CI* confidence interval; *C* + *R* chlorambucil plus rituximab; *CR* complete response; *CRi* complete response with incomplete bone marrow recovery; *DoR* duration of response; *HR* hazard ratio; *nPR* nodular partial response; *NR* not reached; *PR* partial response

**Fig. 4:**
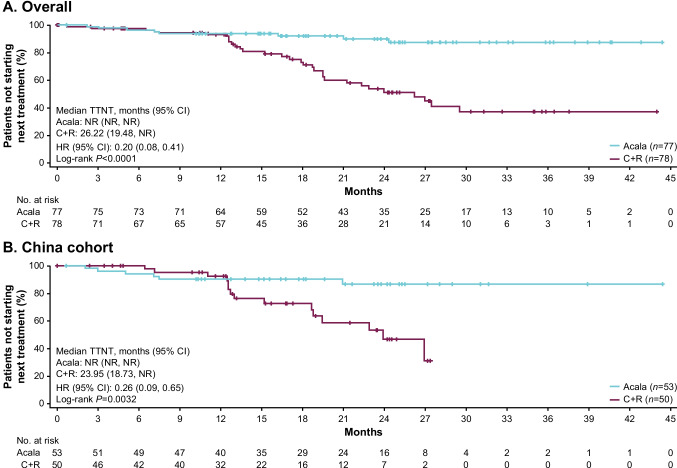
Time to next treatment in (**A**) the overall cohort and (**B**) the China cohort. TTNT was defined as the time from the date of randomization until initiation of non–protocol-specified treatment for CLL or death due to any cause, whichever came first. The median TTNT was calculated using the Kaplan–Meier method. CI for median TTNT was derived based on the Brookmeyer–Crowley method. The analysis was performed using the unstratified log-rank test. The *P*-values are nominal for both cohorts. The HR was calculated using the unstratified Cox model with treatment as the only covariate. The CI for the HR was calculated using the profile likelihood. HR < 1 favors acalabrutinib. *Acala* acalabrutinib; *CI* confidence interval; *CLL* chronic lymphocytic leukemia; *C* + *R* chlorambucil plus rituximab; *HR* hazard ratio; *NR* not reached; *TTNT* time to next treatment

**Fig. 5:**
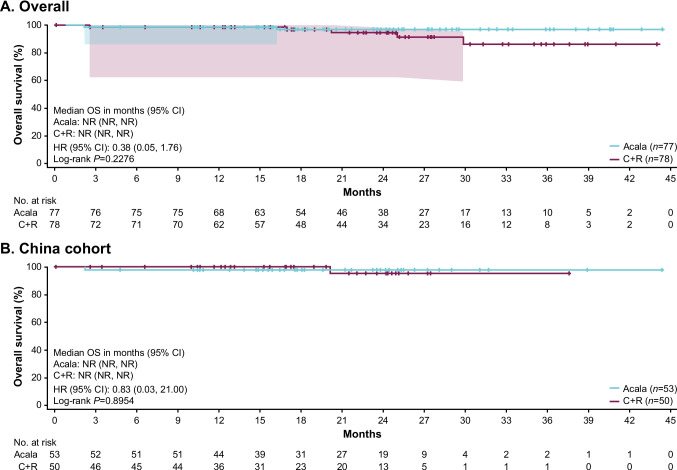
Overall survival in (**A**) the overall cohort and (**B**) the China cohort. Median OS was calculated using the Kaplan–Meier method. CI for median OS was derived based on the Brookmeyer–Crowley method. The analysis was performed using the unstratified log-rank test. The *P*-values are nominal for both cohorts. The HR was calculated using the unstratified Cox model with treatment as the only covariate. The CI for the HR was calculated using the profile likelihood. HR < 1 favors acalabrutinib. *Acala* acalabrutinib; *CI* confidence interval; *C* + *R* chlorambucil plus rituximab; *HR* hazard ratio; *NR* not reached; *OS* overall survival

In the overall cohort, BICR-assessed ORR was 76.6% in the acalabrutinib arm vs 71.8% in the C + R arm (odds ratio [OR], 1.28; 95% CI, 0.62, 2.62; Online Resource 1 Fig. S5A; Table 2). Median BICR-assessed DoR was NR (95% CI, NE, NE) for the acalabrutinib group vs 11.6 months (95% CI, 8.28, 14.26) for the C + R group (HR, 0.05 [95% CI, 0.01, 0.14]; *P* < 0.0001; Fig. 3A). Investigator-assessed ORR was significantly higher with acalabrutinib vs C + R (81.8% vs 60.3%; OR, 2.94 [95% CI, 1.41, 6.11]; *P* = 0.0034) and investigator-assessed DoR (HR, 0.15 [95% CI, 0.05, 0.36]; *P* < 0.0001; Online Resource 1 Table S3) was consistent with BICR assessment. Median TTNT was NR (95% CI, NE, NE) for the acalabrutinib group and 26.2 months (95% CI, 19.48, NE) for the C + R group (HR, 0.20 [95% CI, 0.08, 0.41]; *P* < 0.0001; Fig. 4A). Median OS was NR (95% CI, NE, NE) for both groups (HR, 0.38 [95% CI, 0.05, 1.76]; *P* = 0.2276); Fig. 5A).

**Table 2:** Best overall response and duration of response, BICR assessed (ITT population)

	Overall cohort		China cohort	
	Acala (*n* = 77)	C + R (*n* = 78)	Acala (*n* = 53)	C + R (*n* = 50)
ORR, *n* (%)	59 (76.6)	56 (71.8)	39 (73.6)	35 (70.0)
Odds ratio (95% CI)	1.28 (0.62, 2.62)		1.03 (0.43, 2.46)	
2-sided *P-*value	0.5038		0.9390	
Best overall response, *n* (%)				
CR	3 (3.9)	1 (1.3)	0	1 (2.0)
CRi	0	1 (1.3)	0	1 (2.0)
nPR	0	0	0	0
PR	56 (72.7)	54 (69.2)	39 (73.6)	33 (66.0)
PRL	1 (1.3)	0	1 (1.9)	0
SD	11 (14.3)	11 (14.1)	9 (17.0)	9 (18.0)
PD	0	2 (2.6)	0	2 (4.0)
Unknown	6 (7.8)	9 (11.5)	4 (7.5)	4 (8.0)
Responders who subsequently progressed or died, *n* (%)				
Progression	3 (5.1)	33 (58.9)	2 (5.1)	18 (51.4)
Death without progression	0	0	0	0
Median duration of response, months (95% CI)	NE (NE, NE)	11.56 (8.28, 14.26)	NE (22.01, NE)	10.91 (8.28, 12.52)
Hazard ratio (95% CI)	0.05 (0.01, 0.14)		0.02 (0.00, 0.11)	
Log-rank *P*-value	< 0.0001		< 0.0001	

Unknown includes patients without any adequate post-baseline disease assessment

Responses are assessed by BICR per iwCLL 2018 criteria

Hazard ratio < 1 for duration of response favors acalabrutinib

*BICR* blinded independent central review; *C* + *R* chlorambucil plus rituximab; *CI* confidence interval; *CR* complete response; *CRi* complete response with incomplete bone marrow recovery; *ITT* intention-to-treat; *iwCLL* International Workshop on Chronic Lymphocytic Leukemia; *NE* not evaluable; *nPR* nodular partial response; *ORR* overall response rate; *PR* partial response; *PRL* partial response with lymphocytosis; *SD* stable disease; *PD* progressive disease

In the China cohort, efficacy results were consistent with those of the overall cohort. The median BICR-assessed PFS was significantly longer with acalabrutinib (NR [95% CI, 27.66, NE]) vs C + R (14.4 months [95% CI, 11.53, 16.99]; HR, 0.08 [95% CI, 0.02, 0.22]; *P* < 0.0001; Fig. 2B). BICR-assessed ORR was 73.6% in the acalabrutinib arm and 70.0% in the C + R arm (Online Resource 1 Fig. S5B; Table 2), with significantly longer median DoR in the acalabrutinib arm (NR [95% CI, 22.01 months, NE]) vs the C + R arm (10.9 months [95% CI, 8.28, 12.52]; HR, 0.02 [95% CI, 0.00, 0.11]; *P* < 0.0001; Table 2 and Fig. 3B). Median TTNT was NR (95% CI, NE, NE) for the acalabrutinib group and 23.95 months (95% CI, 18.73, NR) for the C + R group (Fig. 4B). Median OS was NR (95% CI, NR, NR) for both groups in the China cohort (Fig. 5B).

### Safety

A total of 150 patients received ≥ 1 dose of study drug in the overall cohort (acalabrutinib arm, *n* = 77; C + R arm, *n* = 73); in the China cohort, 100 patients received ≥ 1 dose of study drug (acalabrutinib arm, *n* = 53; C + R arm, *n* = 47). The median duration of exposure was similar in the overall and China cohorts (acalabrutinib arm, 23.2 and 19.6 months, respectively; C + R arm, 5.6 and 5.7 months, respectively).

In the overall cohort, treatment-emergent AEs (TEAEs) of any grade were reported in 142 (94.7%) patients (acalabrutinib arm, *n* = 74 [96.1%]; C + R arm, *n* = 68 [93.2%]). In the acalabrutinib arm, COVID-19 was the most common TEAE of any grade, reported in 28 (36.4%) patients; by comparison, COVID-19 was reported in 10 (13.7%) patients in the C + R arm. In the C + R arm, decreased neutrophil count was the most common TEAE of any grade, reported in 19 (26.0%) patients compared with 10 (13.0%) patients in the acalabrutinib arm. Overall, grade ≥ 3 TEAEs were reported in 43 (55.8%) patients in the acalabrutinib arm and 27 (37.0%) in the C + R arm, most commonly increased lymphocyte count in the acalabrutinib arm, reported in 9 (11.7%) patients (vs 0 in the C + R arm), and decreased neutrophil count in the C + R arm, reported in 11 (15.1%) patients (vs 5 [6.5%] in the acalabrutinib arm).

Overall, 32 (41.6%) patients in the acalabrutinib arm and 18 (24.7%) patients in the C + R arm had AEs leading to dose interruptions, most commonly COVID-19 (*n* = 13 [16.9%]) in the acalabrutinib arm and decreased neutrophil count (*n* = 6 [8.2%]) in the C + R arm. No patients in the acalabrutinib arm and 9 (12.3%) patients in the C + R arm had AEs leading to dose reductions, most commonly decreased neutrophil count (*n* = 4 [5.5%]) (Online Resource 1 Table S4). AEs leading to treatment discontinuation occurred in 8 (10.4%) patients in the acalabrutinib arm (hepatitis B reactivation, *n* = 3; breast cancer, decreased platelet count, maculopapular rash, pneumonia, purpura, *n* = 1 each) and 8 (11.0%) patients in the C + R arm (anemia and neutropenia, *n* = 2 each; acute kidney injury, COVID-19 pneumonia, hepatitis B reactivation, tumor lysis syndrome, *n* = 1 each) (Online Resource 1 Table S5). Serious AEs were reported in 30 (39.0%) patients in the acalabrutinib arm and 17 (23.3%) patients in the C + R arm in the overall cohort (Online Resource 1 Table S6). Pneumonia was the most frequent serious AE in both treatment arms (acalabrutinib arm, *n* = 7 [9.1%]; C + R arm, *n* = 4 [5.5%]).

Exposure-adjusted rates per 100 person-years of both treatment-emergent and treatment-related any-grade AEs, grade ≥ 3 AEs, AEs leading to dose modifications, and serious AEs are reported in Online Resource 1 Table S7. The exposure-adjusted AE incidence rates were numerically higher in the C + R arm across the categories.

Ventricular arrhythmias, evaluated as AEs of special interest, were reported in 3 (3.9%) patients in the acalabrutinib arm: ventricular arrhythmia (grade 3; *n* = 1 [1.3%]), ventricular extrasystoles (grade 1 and grade 2; *n* = 1 [1.3%] each), and ventricular tachycardia (grade 3, n = 1 [1.3%]); no ventricular arrhythmias were reported in the C + R arm. Among the predefined events of clinical interest (reported in Table 3 and Online Resource 1 Table S8), no atrial fibrillation or hypertension events were reported in the acalabrutinib arm, and hypertension was reported in 1 (1.4%) patient in the C + R arm. Any-grade ventricular tachyarrhythmias were reported in 3 (3.9%) patients in the acalabrutinib arm and no patients in the C + R arm. Major hemorrhage events were reported in 2 (2.6%) patients in the acalabrutinib arm (grade 2 traumatic intracranial hemorrhage [overall cohort only] and grade 3 purpura [overall and China cohorts]) and no patients in the C + R arm. Infections were reported in 52 (67.5%) patients in the acalabrutinib arm and 27 patients (37.0%) in the C + R arm. Any-grade cardiac and grade ≥ 3 infection events of clinical interest are detailed in Online Resource 1 Tables S9 and S10, respectively.

**Table 3:** Treatment exposure and safety

	Overall cohort				China cohort			
Events	Acala (*n* = 77)		C + R (*n* = 73)		Acala (*n* = 53)		C + R (*n* = 47)	
Treatment exposure median (range), months	23.2 (0–44)		5.6 (1–11)		19.5 (0–44)		5.7 (2–11)	
Any events^a^	TEAE	TRAE^b^	TEAE	TRAE^b^	TEAE	TRAE^b^	TEAE	TRAE^b^
Any AE	74 (96.1)	55 (71.4)	68 (93.2)	51 (69.9)	50 (94.3)	39 (73.6)	45 (95.7)	38 (80.9)
Grade ≥ 3 AE	43 (55.8)	30 (39.0)	27 (37.0)	20 (27.4)	31 (58.5)	23 (43.4)	18 (38.3)	14 (29.8)
Any AE leading to treatment discontinuation	8 (10.4)	4 (5.2)	8 (11.0)	6 (8.2)	6 (11.3)	3 (5.7)	3 (6.4)	3 (6.4)
Any AE leading to dose interruption	32 (41.6)	16 (20.8)	18 (24.7)	15 (20.5)	20 (37.7)	10 (18.9)	13 (27.7)	12 (25.5)
Any AE leading to dose reduction	0	0	9 (12.3)	9 (12.3)	0	0	5 (10.6)	5 (10.6)
Any SAE	30 (39.0)	12 (15.6)	17 (23.3)	12 (16.4)	18 (34.0)	5 (9.4)	10 (21.3)	7 (14.9)
Any AE leading to death	1 (1.3)	0	1 (1.4)	0	1 (1.9)	0	0	0
Events (preferred terms)^a^	Any grade	Grade ≥ 3	Any grade	Grade ≥ 3	Any grade	Grade ≥ 3	Any grade	Grade ≥ 3
COVID-19	28 (36.4)	4 (5.2)	10 (13.7)	0	17 (32.1)	1 (1.9)	8 (17.0)	0
Headache	15 (19.5)	1 (1.3)	6 (8.2)	0	11 (20.8)	1 (1.9)	3 (6.4)	0
Anemia	12 (15.6)	7 (9.1)	12 (16.4)	6 (8.2)	7 (13.2)	4 (7.5)	9 (19.1)	5 (10.6)
Leukocytosis	12 (15.6)	7 (9.1)	1 (1.4)	0	10 (18.9)	6 (11.3)	1 (2.1)	0
Lymphocyte count increased	11 (14.3)	9 (11.7)	0	0	10 (18.9)	8 (15.1)	0	0
Platelet count decreased	11 (14.3)	5 (6.5)	9 (12.3)	4 (5.5)	11 (20.8)	5 (9.4)	9 (19.1)	4 (8.5)
Neutrophil count decreased	10 (13.0)	5 (6.5)	19 (26.0)	11 (15.1)	10 (18.9)	5 (9.4)	17 (36.2)	10 (21.3)
Pneumonia	8 (10.4)	6 (7.8)	6 (8.2)	4 (5.5)	6 (11.3)	5 (9.4)	5 (10.6)	4 (8.5)
COVID-19 pneumonia	5 (6.5)	5 (6.5)	1 (1.4)	0	4 (7.5)	4 (7.5)	0	0
Neutropenia	4 (5.2)	3 (3.9)	11 (15.1)	6 (8.2)	0	0	3 (6.4)	2 (4.3)
White blood cell count decreased	0	0	9 (12.3)	4 (5.5)	0	0	9 (19.1)	4 (8.5)
Events of clinical interest^c^	Any grade	Grade ≥ 3	Any grade	Grade ≥ 3	Any grade	Grade ≥ 3	Any grade	Grade ≥ 3
Cardiac events	7 (9.1)	2 (2.6)	2 (2.7)	0	4 (7.5)	1 (1.9)	0	0
Atrial fibrillation	0	0	0	0	0	0	0	0
Ventricular tachyarrhythmias	3 (3.9)	1 (1.3)	0	0	2 (3.8)	1 (1.9)	0	0
Ventricular extrasystoles	2 (2.6)	0	0	0	1 (1.9)	0	0	0
Ventricular tachyarrhythmia	1 (1.3)	1 (1.3)	0	0	1 (1.9)	1 (1.9)	0	0
Ventricular tachycardia	1 (1.3)	1 (1.3)	0	0	1 (1.9)	1 (1.9)	0	0
Neutropenia	14 (18.2)	8 (10.4)	30 (41.1)	17 (23.3)	10 (18.9)	5 (9.4)	20 (42.6)	12 (25.5)
Hemorrhage	16 (20.8)	1 (1.3)	4 (5.5)	0	12 (22.6)	1 (1.9)	3 (6.4)	0
Major hemorrhage	2 (2.6)	1 (1.3)	0	0	1 (1.9)	1 (1.9)	0	0
Purpura	1 (1.3)	1 (1.3)	0	0	1 (1.9)	1 (1.9)	0	0
Traumatic intracranial hemorrhage	1 (1.3)	0	0	0	0	0	0	0
Hypertension	0	0	1 (1.4)	0	0	0	1 (2.1)	0
Infections	52 (67.5)	17 (22.1)	27 (37.0)	5 (6.8)	34 (64.2)	10 (18.9)	20 (42.6)	4 (8.5)

Data are *n* (%) unless otherwise specified

^a^Patients with multiple events in the same category are counted only once in that category. Patients with events in more than one category are counted once in each of those categories. TEAEs that occurred in ≥ 20% (any grade) and ≥ 5% (grade ≥ 3) of patients in any arm are reported

^b^As assessed by investigator

^c^Patients with multiple events of clinical interest are counted once for each category/subcategory

*AE* adverse event; *C* + *R* chlorambucil plus rituximab; *SAE* serious adverse event; *TEAE* treatment-emergent adverse event; *TRAE* treatment-related adverse event

Deaths are detailed in Online Resource 1 Table S11. A total of 2 (2.6%) deaths, 1 due to sepsis and 1 due to mantle cell lymphoma, neither of which were treatment-related, were reported in the acalabrutinib arm in the overall cohort. A total of 4 (5.5%) deaths were reported in the C + R arm in the overall cohort (sepsis, septic shock, respiratory failure, and hospital-acquired pneumonia; *n* = 1 each), none of which were considered treatment-related.

Safety findings in the China cohort were consistent with those reported in the overall cohort (Table 3 and Online Resource 1 Tables S4‒S6 and S8‒S11).

## Discussion

This randomized, regional, multicenter, open-label study is the first phase 3 study to evaluate the efficacy and safety of acalabrutinib monotherapy in a population of Asian patients with TN CLL. At a median follow-up of 24 months overall and 18 months in the China cohort, significant PFS benefit was observed with acalabrutinib compared with C + R, including in patients with 11q deletions, unmutated IGHV, or complex karyotype. In the overall study cohort, of the 13 patients with complex karyotype who received acalabrutinib, only 1 (7.7%) had a PFS event compared with 6 (40.0%) of 15 patients with complex karyotype who received C + R. The efficacy and safety of acalabrutinib were consistent in the overall and China cohorts.

Efficacy results reported in this Asian population were similar to those reported in the pivotal, global ELEVATE-TN trial in a predominantly North American and European patient population with TN CLL [11]. The ELEVATE-TN population was similar to that of this study except patients with 17p deletion and/or *TP53* mutation were included in ELEVATE-TN (comprising 14% of patients) and excluded in the present study. In the ELEVATE-TN trial, patients with TN CLL were randomized to receive either treat-to-progression acalabrutinib with or without 6-month treatment with obinutuzumab, or 6-month treatment with chlorambucil plus obinutuzumab. At a median follow-up of 28.3 months, patients receiving acalabrutinib monotherapy had comparable ORR (86%), estimated 24-month PFS rate (87%), and median PFS (NR) to patients in this study. In both ELEVATE-TN and the current trial, a significant PFS benefit in favor of acalabrutinib monotherapy over chlorambucil plus an anti-CD20 antibody was observed, with significant risk reductions of 80% (ELEVATE-TN) and 92% (ChangE). The use of the type I anti-CD20 antibody, rituximab, in the comparator arm of ChangE could have contributed to higher risk reduction relative to ELEVATE-TN.

The phase 3 SEQUOIA and RESONATE-2 studies also compared covalent BTKi monotherapy with fixed-duration CIT in frontline CLL, providing context to these findings [18, 19]. In the SEQUOIA study, patients with TN CLL received either the second-generation BTKi zanubrutinib or bendamustine plus rituximab. At a median follow-up of 26.2 months, patients without del(17p) who received zanubrutinib monotherapy had an ORR including partial response with lymphocytosis of 95% and an estimated 24-month PFS rate of 86%; median PFS was NR. In the RESONATE-2 study, patients with TN CLL received either the first-generation BTKi ibrutinib or chlorambucil, and at a median follow-up of 29 months, patients receiving ibrutinib had an ORR of 92%, an estimated 24-month PFS rate of 89%, and a median PFS of NR. The median age of patients receiving BTKi was ≥ 70 years for patients in both studies, suggesting a similar patient population between all 3 studies. Both studies showed a significant PFS benefit of BTKi therapy vs comparator with significant risk reductions of 58% seen with zanubrutinib in the SEQUOIA study and 88% seen with ibrutinib in RESONATE-2. Similarly, ibrutinib monotherapy and ibrutinib-venetoclax demonstrated PFS benefit vs fixed-duration CIT (fludarabine-cyclophosphamide-rituximab) in the FLAIR study [20] and the fixed-duration combinations venetoclax-obinutuzumab and venetoclax-ibrutinib demonstrated noninferior PFS vs ibrutinib monotherapy in the CLL17 study [21]. Together, these findings demonstrate the benefit of BTKi-based therapy vs CIT or chemotherapy and the noninferiority of chemotherapy-free fixed-duration combinations vs treat-to-progression BTKi therapy, which potentially expands treatment options for patients with TN CLL.

In comparing our findings with those of other studies of BTKis in Asian populations, patients in the overall acalabrutinib cohort in the present study had a somewhat lower ORR (76.6%) compared with patients with treatment-naive CLL who received zanubrutinib (96.4%) in a real-world study in China at a similar median follow-up (23.5 months vs 21.8 months, respectively) [22]. However, the estimated 24-month PFS rate (92%) in patients receiving acalabrutinib in our study was higher than that reported in the real-world study for zanubrutinib (83%), and the rate of acalabrutinib treatment discontinuation (10%) was lower than that of zanubrutinib (15%). In addition, patients in the acalabrutinib arm had similar ORR and a numerically higher estimated 24-month PFS rate compared with Japanese patients with treatment-naive CLL who received ibrutinib in a real-world study (ORR, 76.6%; estimated 24-month PFS rate, 85%), even with a higher percentage of patients with Ann Arbor or Rai stage III–IV disease (57% vs 46%) [23]. It should be noted that comparisons between ChangE and other clinical trials or real-world studies have fundamental limitations, including differing study designs and patient populations. Head-to-head studies are required to evaluate differences in efficacy between acalabrutinib and other BTKis.

Acalabrutinib was well tolerated, and TEAEs were manageable. Rates of AEs were consistent across cohorts, and reported AEs were consistent with the known safety profile of acalabrutinib. Regarding cardiac safety, 3 patients treated with acalabrutinib experienced ventricular tachyarrhythmia (grade 1─2, *n* = 2; grade ≥ 3, *n* = 1). No events of atrial fibrillation or hypertension of any grade were observed with acalabrutinib in this study, which is consistent with the low rates of these ECIs reported in ELEVATE-TN (4% and 2%, respectively) [11]. Infection rates were higher with acalabrutinib (any grade [grade ≥ 3]: 67.5% [22.1%]) vs C + R (37.0% [6.8%]) in this study, consistent with the findings from ELEVATE-TN (acalabrutinib: 65.4% [14.0%]; CIT: 43.8% [8.3%]) [11]. The most common serious infections in this study were COVID-19 and COVID-19 pneumonia in both treatment arms. There were no unexpected safety observations in the overall cohort or the China cohort in ChangE.

Study limitations include that the C + R comparator regimen used in this study is no longer recommended for all patients with TN CLL [24], which limits the interpretation of treatment benefit relative to current standards of care. In addition, the study had insufficient follow-up to discern differences in OS between acalabrutinib and C + R and to assess long-term safety. The crossover design may also preclude development of any significant differences in OS, even with longer follow-up. A total of 5 patients randomized to the C + R arm did not receive study treatment, which also may have affected comparisons with the acalabrutinib arm for the intention-to-treat population. There was also a higher number of early withdrawals in the C + R arm, with most occurring prior to treatment initiation; however, this likely had a minimal impact on study findings due to the magnitude of difference in PFS between the treatment arms. Additionally, real-world studies in China and Asia must also be conducted to determine whether the recent approval of acalabrutinib will lead to increased benefit in clinical practice in these countries. Although the study was conducted during the COVID-19 pandemic in China, the outbreak of COVID-19 was not considered to have had a significant impact on the study.

Acalabrutinib demonstrated a statistically significant improvement in PFS vs C + R in patients with TN CLL in the overall and China cohorts. These results, in a population of patients from across Asia, are consistent with results from other global studies showing that acalabrutinib is an effective and well-tolerated treatment for patients with TN CLL.

## Supplementary Information

Below is the link to the electronic supplementary material.

Supplementary file1 (PDF 831 KB)

## Data Availability

Data underlying the findings described in this article may be obtained in accordance with AstraZeneca’s data sharing policy described at (https://astrazenecagrouptrials.pharmacm.com/ST/Submission/Disclosure). Data for studies directly listed on Vivli can be requested through Vivli at (http:/www.vivli.org). Data for studies not listed on Vivli can be requested through Vivli at (https://vivli.org/members/enquiries-about-studies-not-listed-on-the-vivli-platform/). AstraZeneca Vivli member page is also available outlining further details: (https://vivli.org/ourmember/astrazeneca/).
