# Supplementary material for: Acalabrutinib vs chlorambucil plus rituximab in untreated chronic lymphocytic leukemia: A randomized phase 3 Asian study
Source: Ann Hematol. 2026 May 19;105(8):327. doi: 10.1007/s00277-026-07006-z (PMC13357647; doi:10.1007/s00277-026-07006-z)
Supplement: Supplementary file 1 — Supplementary file1 (PDF 831 KB) [file 277_2026_7006_MOESM1_ESM.pdf]

ONLINE RESOURCE 1

Acalabrutinib vs chlorambucil plus rituximab in untreated chronic lymphocytic leukemia: A randomized phase 3 Asian study

Annals of Hematology

Jia Li*,[1] Shuhua Yi*,[2,3] Thanh Nguyen,[4] Wenjuan Yu,[5] Keshu Zhou,[6] Haiwen Huang,[7] Udomsak Bunworasate,[8] Man Huynh,[9] Hui Zhou,[10] Su-Peng Yeh,[11] Yiqiu Wang,[12] Xiaofeng Liu,[12] Wei Fu,[12] Lugui Qiu[2,3]

*These authors contributed equally to this work.
[1]Department of Hematology, Shengjing Hospital of China Medical University, Shenyang, China; [2]State Key Laboratory of Experimental Hematology, National Clinical Research Center for Blood Diseases, Haihe Laboratory of Cell Ecosystem, Institute of Hematology & Blood Diseases Hospital, Chinese Academy of Medical Sciences & Peking Union Medical College, Tianjin, China; [3]Tianjin Institutes of Health Science, Tianjin, China; [4]National Institute of Hematology and Blood Transfusion, Hanoi, Vietnam; [5]Department of Hematology, The First Affiliated Hospital, Zhejiang University College of Medicine, Hangzhou, China; [6]Department of Hematology, The Affiliated Cancer Hospital of Zhengzhou University, Henan Cancer Hospital, Zhengzhou, China; [7]The First Hospital of Soochow University, Suzhou, China; [8]Department of Medicine, Faculty of Medicine, Chulalongkorn University, Bangkok, Thailand; [9]Blood Transfusion Hematology Hospital, Ho Chi Minh City, Vietnam; [10]Department of Lymphoma & Hematology, Hunan Cancer Hospital, The Affiliated Cancer Hospital of Xiangya School of Medicine, Central South University, Changsha, China; [11]Division of Hematology and Oncology, Department of Internal Medicine, China Medical University Hospital, Taichung, Taiwan; [12]AstraZeneca, Shanghai, China

Address for correspondence:
Lugui Qiu, MD
qiulg@ihcams.ac.cn

Table of Contents

Plain language summary	2
List of ChangE primary investigators by country/region and study site	3
**Supplementary Methods**	6
Detailed inclusion/exclusion criteria	6
Dose modification and AE management guidelines	11
Definitions of endpoints and additional details of statistical analyses	14
**Supplementary Figures**	
Figure S1. Kaplan-Meier plot of progression-free survival in the overall cohort (investigator-assessed) (ITT population)	18
Figure S2. Progression-free survival by BICR, subgroup analysis (ITT population)	19
Figure S3. Kaplan-Meier plot of progression-free survival (BICR-assessed) by treatment and 11q deletion (ITT population)	20
Figure S4. Kaplan-Meier Plot of progression-free survival (BICR-assessed) by treatment and IGHV status (ITT population)	21
Figure S5. BICR-assessed response rates in the overall cohort and the China cohort	22
**Supplementary Tables**	
Table S1. Demographics and baseline characteristics (ITT population)	23
Table S2. Summary of COVID-19 study disruptions (ITT population)	25
Table S3. Best overall response and duration of response, assessed by investigator (ITT population)	26
Table S4. AEs leading to dose interruption or reduction of any treatment by system organ class and preferred term (safety population)	27
Table S5. AEs leading to treatment discontinuation by system organ class and preferred term (safety population)	30
Table S6. Serious AEs by system organ class and preferred term (n>1 in combined treatment arms) (safety population)	32
Table S7. Exposure-adjusted AEs in any category (safety population)	35
Table S8. Overall summary of events of clinical interest (safety population)	36
Table S9. Cardiac events (any grade and grade ≥3) by preferred term (safety population)	38
Table S10. Grade ≥3 infections by preferred term (n>1 event in combined treatment arms) (safety population)	39
Table S11. Deaths (ITT population)	40
Reference	41

Plain language summary

Chronic lymphocytic leukemia, or CLL, is a rare type of cancer that leads to the over-production of blood cells. Acalabrutinib is an oral drug that inhibits the activity of a protein called Bruton tyrosine kinase, which is important in the development of CLL. Acalabrutinib has been approved for the treatment of CLL in multiple countries, including China, for patients with CLL whose disease returned or did not respond to other therapy. This study investigated patient responses (efficacy) and side effects (safety) to acalabrutinib compared with the standard combination of chlorambucil (a type of chemotherapy) and rituximab (a type of therapy that uses the body's own defenses, ie, the immune system, to fight cancer), in patients with untreated CLL across Asia, including China. Patients older than 65 years of age, or younger patients (aged 18 to 65 years) with more serious disease characteristics, were randomly assigned to receive either acalabrutinib or the chlorambucil plus rituximab. Efficacy and safety results were collected throughout the course of the study. The study included 155 patients from sites across Asia, including mainland China, Taiwan, Vietnam, Thailand, and the Philippines. At 24 months of follow-up on acalabrutinib, 92% of patients were alive and had no disease progression (compared with 25% of patients treated with the chlorambucil plus rituximab combination). There were no new side effects found in patients who received acalabrutinib. These results, in a population of patients from Asia, confirm the results from other global studies showing that acalabrutinib is an effective and well-tolerated treatment for patients with previously untreated CLL. These results can inform treatment decisions for patients from Asia with previously untreated CLL.

List of ChangE Primary Investigators by Country/Region and Study Site

Country	Investigator (site)
Mainland China	Shuhua Yi (Institute of Hematology & Blood Diseases Hospital, Chinese Academy of Medical Sciences), Jishi Wang (Affiliated Hospital Guiyang Medical College), Kaiyang Ding (Anhui Provincial Cancer Hospital), Keshu Zhou (Henan Cancer Hospital), Jie Ma (The First Affiliated Hospital of Zhengzhou University), Jie Jin (Zhejiang University), Wenjuan Yu (Zhejiang University), Fei Li (The First Affiliated Hospital of Nanchang University), Zhuogang Liu (The Second Hospital affiliated to China Medical University), Haiwen Huang (The First Affiliated Hospital of Suzhou University), Sujun Gao (The First Hospital of Jilin University), Ru Feng (Nanfang Hospital), Jianqing Mi (Ruijn Hospital Affiliated to Shanghai Jiaotong University), Tong Chen (Fudan University), Lihong Liu (The Fourth Hospital of Hebei Medical University, Hebei Tumor Hospital), Wei Wang (The Affiliated Hospital of Qingdao University), Linhua Yang (The Second Hospital of Shanxi Medical University), Hui Zhou (Hunan Cancer Hospital)
Philippines	Kimberley Cutiyog-Ubando (Baguio General Hospital & Medical Center), Rosalio Torres (Makati Medical Center), Jay Datukan (St. Luke's Medical Center QC), Teresita Dumagay (Philippine General Hospital), Hiede Abdurahman (Metro Davao Medical and Research Center), Maria Christine Joy Sarmiento (Perpetual Succour Hospital)
Taiwan	Chun-Kai Liao (Kaohsiung Chang Gung Memorial Hospital), Hung-Lin Liu (Kaohsiung Chang Gung Memorial Hospital), Ming-Chung Wang (Kaohsiung Chang Gung Memorial Hospital), Tsai-Yun Chen (National Cheng-Kung University Hospital), Liang-Tsai Hsiao (Veteran General Hospital Taipei), Su-Peng Yeh (China Medical University Hospital), Shang-Ju Wu (National Taiwan University Hospital), Chieh-Lin Teng (Veterans General Hospital Taichung)
Thailand	Udomsak Bunworasate (King Chulalongkorn Memorial Hospital, Chulalongkorn University), Archrob Khuhapinant (Siriraj Hospital, Mahidol University), Lalita Norasetthada (Maharaj Nakorn Chiang Mai Hospital), Kanchana Chansung (Srinagarind Hospital, Khon Kaen University), Nattiya Teawtrakul (Srinagarind Hospital, Khon Kaen University), Kannadit Prayongratana (Phramongkutklao Hospital), Tawatchai Suwanban (Rajavithi Hospital),

	Daolada Kongkabpan (Songklanagarind Hospital, Prince of Songkla University)
Vietnam	Thanh Nguyen (National Institute of Hematology & Blood Transfusion), Man Huynh (Blood Transfusion - Hematology Hospital), Tung Tran (Cho Ray Hospital)

Supplementary Methods

Detailed Inclusion/Exclusion Criteria

Inclusion Criteria

Informed consent

- Capable of giving signed informed consent, which includes compliance with the requirements and restrictions listed in the informed consent form and in the protocol.
- Provision of signed and dated written informed consent form prior to any mandatory study specific procedures, sampling, and analyses.

Age

- Men and women:
  - (a) ≥65 years of age **OR**
  - (b) >18 and <65 years of age, provided that they meet ≥1 of the following criteria:
    - (i) Creatinine clearance 30 to 69 mL/min using the Cockcroft-Gault equation (International Workshop on CLL [iwCLL] guidelines[1]).
    - (ii) A score higher than 6 on the Cumulative Illness Rating Scale-Geriatric.

Type of patient and disease characteristics

- Eastern Cooperative Oncology Group performance status (ECOG PS) of 0, 1, or 2.
- Diagnosis of chronic lymphocytic leukemia (CLL) that meets published diagnostic criteria[1]:
  - (a) Monoclonal B-cells (either kappa or lambda light chain restricted) that are clonally co-expressing B-cell markers (CD19, CD20, and CD23) and CD5.
  - (b) Prolymphocytes may comprise <55% of blood lymphocytes.
  - (c) Presence of $\geq 5\times10^9$ B lymphocytes/L (5000/μL) in the peripheral blood (at any point since the initial diagnosis).
- Active disease per iwCLL 2018 criteria[1] that requires treatment, ≥1 of the following criteria should be met:
  - (a) Evidence of progressive marrow failure as manifested by the development or worsening of anemia (hemoglobin <10 g/dL) and/or thrombocytopenia (platelets <100,000/μL).
  - (b) Massive (ie, ≥6 cm below the left costal margin), progressive, or symptomatic splenomegaly.
  - (c) Massive-node (ie, ≥10 cm in the longest diameter), progressive, or

symptomatic lymphadenopathy.

(d) Progressive lymphocytosis with an increase of >50% over a 2-month period or a lymphocyte doubling time (LDT) of <6 months. LDT may be obtained by linear regression extrapolation of absolute lymphocyte count obtained at intervals of 2 weeks over an observation period of 2 to 3 months. In patients with initial blood lymphocyte counts of <30×10^9/L (30,000/μL), LDT should not be used as a single parameter to define indication for treatment. In addition, factors contributing to lymphocytosis or lymphadenopathy other than CLL (eg, infections) should be excluded.

(e) Autoimmune anemia and/or thrombocytopenia that is poorly responsive to standard therapy.

(f) Symptomatic or functional extranodal involvement (eg, skin, kidney, lung, spine).

(g) B-symptoms documented in the patient's chart with supportive objective measures, as appropriate, defined as ≥1 of the following disease-related symptoms or signs:

    (i) Unintentional weight loss ≥10% within the previous 6 months before screening.

    (ii) Significant fatigue (ECOG PS 2 or higher; inability to work or perform usual activities).

    (iii) Fevers higher than 100.5°F or 38.0°C for ≥2 weeks before screening without evidence of infection.

    (iv) Night sweats for ≥1 month before screening without evidence of infection.

- Meet the following laboratory parameters:

  (a) Adequate bone marrow function independent of growth factor or transfusion support 1 week before assessment, as follows:

      (i) Absolute neutrophil count (ANC) ≥750 cells/μL (0.75×10^9/L); ANC ≥500 cells/μL (0.50×10^9/L) in patients with documented bone marrow involvement of CLL.

      (ii) Platelet count ≥50,000 cells/μL (50×10^9/L); platelet count ≥30,000 cells/μL (30×10^9/L) in patients with documented bone marrow involvement of CLL.

  (b) Serum aspartate aminotransferase and alanine aminotransferase ≤2.5× upper limit of normal (ULN).

  (c) Total bilirubin ≤2.0×ULN, unless directly attributable to Gilbert's syndrome.

  (d) Estimated creatinine clearance of ≥30 mL/min using the Cockcroft-Gault

equation.

Reproduction

- Negative pregnancy test (urine or serum) for female patients of childbearing potential prior to enrollment.
- Prior to the planned date of randomization for female patients, ≥1 of the following criteria should be met:
  (a) ≥1 year post-menopausal.
  (b) Permanently sterilized (hysterectomy, bilateral oophorectomy, or bilateral salpingectomy).
  (c) Or using highly effective methods of contraception for the duration of the study (from the time they sign consent) and for 2 days after the last dose of acalabrutinib or 12 months after the last dose of rituximab or chlorambucil, whichever is longer, to prevent pregnancy.
- For rituximab and chlorambucil: male patients who are sexually active must agree to use highly effective methods of contraception with the addition of a barrier method (condom) during the study (from the time they sign consent) and for 90 days after the last dose of rituximab or chlorambucil, whichever is later, to prevent pregnancy in a partner. Male patients must agree to refrain from sperm donation during this same time period.

Exclusion criteria

Medical conditions

- Known detected del(17p) or *TP53* mutation (note: test results should be obtained from central lab during screening).
- Transformation of CLL to aggressive non-Hodgkin lymphoma (eg, Richter's transformation, prolymphocytic leukemia, or diffuse large B-cell lymphoma) or central nervous system involvement by leukemia.
- History of confirmed progressive multifocal leukoencephalopathy.
- History of prior malignancy that could affect compliance with the protocol or interpretation of results, except for the following:
  (a) Curatively treated basal cell carcinoma or squamous cell carcinoma of the skin or carcinoma in situ of the cervix at any time prior to study.
  (b) Other cancers not specified above which have been curatively treated by surgery and/or radiation therapy from which patient is disease-free for ≥3 years without further treatment.
- Significant cardiovascular disease such as uncontrolled or untreated

symptomatic arrhythmias, congestive heart failure, or myocardial infarction within 6 months of screening, or any class 3 or 4 cardiac disease as defined by the New York Heart Association Functional Classification at Screening. Exception: patients with controlled, asymptomatic atrial fibrillation during screening are allowed to enroll on study.

- Malabsorption syndrome, disease significantly affecting gastrointestinal function, resection of the stomach, or extensive small bowel resection that is likely to affect absorption, symptomatic inflammatory bowel disease, or partial or complete bowel obstruction, or gastric restrictions and bariatric surgery, such as gastric bypass.
- Known history of infection with human immunodeficiency virus.
- Serologic status reflecting active hepatitis B or C infection:
  - (a) Patients who are hepatitis B core antibody positive and who are hepatitis B surface antigen (HBsAg) negative will need to have a negative polymerase chain reaction (PCR) result before randomization and must be willing to undergo DNA PCR testing during the study. Those who are HBsAg-positive or hepatitis B PCR-positive will be excluded.
  - (b) Patients who are hepatitis C antibody positive will need to have a negative PCR result before randomization. Those who are hepatitis C PCR-positive will be excluded.
- Any active systemic infection (eg, bacterial, viral, or fungal infection) requiring systemic treatment.
- Uncontrolled autoimmune hemolytic anemia or idiopathic thrombocytopenic purpura, defined as declining hemoglobin or platelet count secondary to autoimmune destruction within the screening period.
- History of bleeding diathesis (eg, hemophilia, von Willebrand disease).
- History of stroke or intracranial hemorrhage within 6 months before first dose of study drug.
- Major surgical procedure within 30 days of first dose of study drug. Note: If a patient had major surgery, they must have recovered adequately from any toxicity and/or complications from the intervention before the first dose of study drug.

Prior/concomitant therapy

- Any prior CLL-specific therapies (note: prior localized radiotherapy is allowed).
- Corticosteroid use >20 mg/day within 1 week before first dose of study drug, except as indicated for other medical conditions such as inhaled steroid for

asthma, topical steroid use, or as premedication for administration of study drug. For example, patients requiring steroids at daily doses >20 mg prednisone equivalent systemic exposure daily, or those who are administered steroids for leukemia control or white blood cell count lowering are excluded.

- Requires or receiving anticoagulation with warfarin or equivalent vitamin K antagonists.
- Requires treatment with a strong CYP3A inhibitor. The use of strong or moderate CYP3A inhibitors or inducers within 7 days of the first dose of study drug is prohibited.
- Received any investigational drug within 30 days before first dose of study drug.
- Received a live virus vaccination within 28 days of first dose of study drug.
- History of known hypersensitivity or anaphylactic reactions to study drugs or excipients.

Prior/concurrent clinical study experience

- Concurrent participation in another therapeutic clinical trial.

Other exclusions

- For women only: breastfeeding or pregnant.
- Involvement in the planning and/or conduct of the study (applies to both sponsor staff and/or staff at the study site).
- Judgment by the investigator that the patient should not participate in the study if the patient is unlikely to comply with study procedures, restrictions, and requirements.

Supplementary Methods

Dose Modification and AE Management Guidelines

Acalabrutinib

The actions in the Acalabrutinib Dose Modification Table should be taken for the following toxicities (according to Common Terminology Criteria for Adverse Events [CTCAE] criteria version 5.0):

- Grade 4 neutropenia (ANC <500/µL) for >7 days (myeloid growth factors are permitted per Consensus of Chinese Experts [version 2015] and use must be recorded on the electronic case report form [eCRF]).
- Grade 3 platelets decreased in the presence of clinically significant bleeding.
- Grade 4 platelets decreased.
- Grade 3 or 4 nausea, vomiting, or diarrhea, if persistent despite optimal antiemetic and/or anti-diarrheal therapy.
- Any other grade 4 toxicity or unmanageable grade 3 toxicity.

If the toxicity resolves or reverts to CTCAE grade 1 or baseline within 28 days of onset and the patient is showing clinical benefit, treatment with acalabrutinib may be restarted using the rules below for dose modifications (see Acalabrutinib Dose Modification Table). Whenever possible, any dose adjustment of acalabrutinib should be discussed between the investigator and the sponsor before implementation. The appropriate clinic staff should dispense the study drug for the new dose level and instruct the patient/caregiver about the change in dose level. Any changes to the dosing regimen must be recorded in the appropriate eCRF.

Once de-escalation has occurred, the dose should not restart at original dose level. Treatment with acalabrutinib should be held for any unmanageable, potentially study drug–related toxicity that is grade ≥3 in severity. Any other clinically important events where dose delays may be considered appropriate by the investigator must be discussed with the sponsor.

Study drug may be held for a maximum of 28 consecutive days from expected dose. Study treatment should be discontinued in the event of a toxicity requiring the postponement of dosing lasting >28 days, unless reviewed and approved by the sponsor.

Temporary withholding of study drug for as little as 7 days can cause a transient worsening of disease and/or of constitutional symptoms.

Acalabrutinib Dose Modification Table

Occurrence	Action
1st–2nd	Hold acalabrutinib until recovery to grade 1 or baseline; may restart at original dose level (100 mg BID).
3rd	Hold acalabrutinib until recovery to grade 1 or baseline; restart at one dose level lower (100 mg QD).
4th	Discontinue acalabrutinib.
BID, twice daily; QD, once daily.	

Rituximab and chlorambucil

If a patient experiences any grade ≥2 nonhematological toxicity, chlorambucil and/or rituximab should be held until the nonhematological toxicity returns to grade 1 or baseline. If treatment is delayed for >4 weeks due to study drug–related toxicity, chlorambucil and/or rituximab should be discontinued.

If a patient experiences grade 3 or 4 cytopenia, the guidelines for dose delay (chlorambucil or rituximab) and dose reduction (chlorambucil only) are outlined in the Rituximab and Chlorambucil Dose Modification Table below.

Rituximab and Chlorambucil Dose Modification Table

	Chlorambucil	Rituximab
Grade 3 or 4 cytopenia	Delay dosing for a maximum of 4 weeks. Administer G-CSF for neutropenia or platelets or red blood cells as required.<br><br>1st episode: If improvement to grade ≤2 (or baseline), decrease Clb dose to 75% of initial dose for subsequent cycles.<br>2nd episode: If improvement to grade ≤2 (or baseline), decrease Clb dose to 50% of initial dose for subsequent cycles.<br>3rd episode: Discontinue Clb.	If improvement to grade ≤2 (or baseline), administer full dose.<br>If Clb is discontinued, rituximab may continue at the investigator's discretion.
Grade 1 or 2 cytopenia	No dose reduction or delay	No dose reduction or delay.
Clb, chlorambucil; G-CSF, granulocyte colony-stimulating factor.		

No reduction in the dose of rituximab is allowed. Severe, including fatal, infusion reactions can occur with rituximab. Discontinue rituximab infusion and provide medical

treatment for grade 3 or 4 infusion reactions. For less severe infusion reactions (grade 1 or 2), interrupt the infusion or slow the infusion rate. Rituximab may continue at the discretion of the investigator when the toxicity has improved (grade ≤2 for hematological toxicity and grade 1 or baseline for nonhematological toxicity). If rituximab is discontinued, the patient is withdrawn from study treatment. Refer to the rituximab local prescribing information for dose withholding or discontinuation in response to specific toxicities associated with rituximab.

Once reduced, the dose of chlorambucil should not be escalated. A delay of up to 4 weeks is permitted for chlorambucil to allow recovery of hematologic toxicities to grade ≤2 or nonhematologic toxicities to grade 1 or baseline level. If the treatment is delayed for more than 4 weeks due to toxicity, chlorambucil should be discontinued.

If a grade 3 or 4 cytopenia prevents treatment on day 15 (of any cycle for arm B), the day 15 chlorambucil dose will be skipped in order to keep the antibody on schedule. Rituximab + chlorambucil administration on day 1 of the following cycle will be given if the cytopenia has resolved to grade ≤2. Chlorambucil will be given at a reduced dose. If the cytopenia persists, rituximab + chlorambucil administration will be delayed until the cytopenia has improved to grade ≤2.

Chlorambucil has been reported to exacerbate or precipitate autoimmune hemolytic anemia and patients should be monitored carefully for this condition. If a rapid decrease of hemoglobin occurs during therapy, the possibility of chlorambucil- or autoantibody-induced hemolysis should be considered and appropriate diagnostic tests (lactate dehydrogenase, bilirubin, haptoglobin, reticulocytes, Coombs test) should be performed. If hemolysis is suspected, a Coombs test should be performed. If, in the judgment of the treating physician, there is evidence of clinically significant hemolytic anemia secondary to chlorambucil, study treatment should be promptly withdrawn. Full details of the hemolytic anemia should be recorded on the adverse event pages of the eCRF.

As many of these patients have multiple comorbidities, treatment may be delayed longer than 4 weeks (for both treatments) to enable resolution of unrelated adverse events (AEs), concurrent diseases, or recovery from surgical procedures. This is at the investigator's discretion but should be discussed in advance with the sponsor.

A patient should discontinue study treatment with chlorambucil/rituximab if any of the following occur:

- Grade 4 infusion-related symptom (patient should be withdrawn immediately).
- Grade 3 infusion-related symptom at re-challenge.

- Grade 3 or 4 cytopenia that has not resolved to grade ≤2 and delays treatment by 4 weeks.
- Grade ≥2 noncytopenic toxicity that does not resolve to grade 1/baseline and delays treatment by 4 weeks.

Definitions of Endpoints and Additional Details of Statistical Analyses

Progression-free survival (PFS)

PFS was defined as the time from the date of randomization until disease progression (assessed by blinded independent central review [BICR] per iwCLL 2018 criteria) or death from any cause, whichever occurred first. Patients who withdrew from the study or were considered lost to follow-up without prior documentation of disease progression were censored on the date of the last adequate disease assessment.

Patients who started new anticancer therapy before documentation of disease progression were censored on the date of the last adequate disease assessment occurring on or before the start date of the new anticancer therapy. For patients without an adequate post-baseline disease assessment, PFS was censored on the date of randomization unless death occurred within 2 visits of baseline.

PFS was also assessed using the same derivation rule described above based on investigator assessment.

The primary analysis was performed in the intention-to-treat (ITT) population to compare PFS as assessed by BICR using a 2-sided stratified log rank test, adjusting for ECOG PS (0–1 vs 2) and Rai stage (0–II vs III–IV) at randomization. The estimate of the hazard ratio (HR) and its corresponding 95% confidence interval (CI) was computed using a Cox proportional-hazards model stratified by the randomization strata.

Kaplan-Meier plots of PFS were presented by treatment arm. Summaries of the number and percentage of patients experiencing a PFS event and the type of event (progression or death) were provided along with median PFS for each treatment.

Subgroup analyses compared PFS between arm A vs arm B in the following subgroups:

- Age at randomization (<65 vs ≥65)
- Sex (male vs female)
- ECOG PS (0–1 vs 2)
- IGHV mutation status (mutated vs nonmutated)
- Country (China vs other)

- Rai stage at screening (0–II vs III–IV)
- β2-microglobulin at baseline (≤3.5 mg/L vs >3.5 mg/L)
- Bulky disease (longest diameter of lymph node <5 cm vs ≥5 cm at baseline)

The HR and corresponding 95% CI for each subgroup were calculated based on an unstratified Cox regression model and presented in a forest plot only for the PFS endpoint using the BICR data (given the absence of a marked difference between the results of the statistical analyses of the PFS from the BICR data and that of the site investigator tumor data).

No adjustment to the significance level for testing of the subgroups was made, as these analyses were supportive of the PFS analysis.

Overall response rate (ORR)

ORR was defined as the proportion of patients who have a complete response (CR), complete response with incomplete bone marrow recovery (CRi), nodular partial response (nPR) or partial response (PR) assessed by BICR per iwCLL 2018 criteria[1] at or before initiation of subsequent anticancer therapy. CRi refers to patients who fulfill all the criteria for a CR (including the bone marrow examinations), but have a persistent anemia, thrombocytopenia, or neutropenia apparently unrelated to CLL, but related to drug toxicity. nPR refers to patients who fulfill all the criteria for a CR but with the presence of B-lymphoid nodules in the bone marrow reflecting residual disease.

Best overall response (BOR) is the best response a patient has after the randomization date but prior to starting any subsequent anticancer therapy, up to and including progression or the last evaluable assessment in the absence of progression.

ORR and BOR were obtained using the same derivation rule described above based on investigator assessment.

ORR (assessed by BICR per iwCLL 2018 criteria[1]) was compared between treatment arms A vs B using the Cochran-Mantel-Haenszel chi-square test, adjusted for randomization stratification factors.

Number and percentage of patients with a tumor response (CR, CRi, nPR, or PR) were presented by treatment arm. For each treatment arm, BOR was summarized by n (%) of each category (CR, CRi, nPR, PR, stable disease, progressive disease, not evaluable). No formal statistical analysis was planned for BOR.

ORR and BOR assessed by investigator were analyzed in the same fashion as that for BICR-assessed ORR and BOR.

Duration of response (DoR)

DoR (assessed by BICR per iwCLL 2018 criteria[1]) was defined as the time from the date of first documented response until date of documented progression or death in the absence of disease progression. Patients who withdrew from the study or were considered lost to follow-up without prior documentation of disease progression were censored on the date of the last adequate disease assessment. Patients who started new anticancer therapy before documentation of disease progression were censored on the date of the last adequate disease assessment occurring on or before the start date of the new anticancer therapy.

DoR was obtained using the same derivation rule described above based on investigator assessment.

DoR assessed by BICR or investigator per iwCLL 2018 criteria[1] was analyzed in the same fashion as that for primary efficacy endpoint PFS.

Time to next therapy (TTNT)

TTNT was defined as time from randomization until institution of non–protocol-specified treatment for CLL or death due to any cause, whichever came first.

TTNT was analyzed in the same fashion as that for primary efficacy endpoint PFS.

Overall survival (OS)

OS was defined as the length of time from randomization until the date of death due to any cause. Any patient not known to have died at the time of analysis was censored based on the last recorded date on which the patient was known to be alive.

OS was analyzed in the same fashion as that for primary efficacy endpoint PFS.

A sensitivity analysis was also performed in which patients who received crossover therapy were censored at the date before first dose date of the crossover therapy.

Safety

Treatment-emergent AEs

The following events are considered treatment emergent:

- AEs with an onset date on or after first dose of study drug, and within 30 days after last dose of study drug.
- Worsening of pre-existing events on or after first dose of study drug, and within 30 days after last dose of study drug.

Safety and tolerability were assessed in terms of AEs (including serious AEs), laboratory data, vital signs, electrocardiograms, and exposure.

“On treatment” was defined as assessments between date of first dose and 30 days following treatment discontinuation.

Fig. S1. Investigator-assessed PFS in the overall cohort. Median PFS was calculated using the Kaplan-Meier technique. Progression is determined by BICR per iwCLL 2018 criteria.[1] CI for median PFS was derived based on Brookmeyer-Crowley method. The analysis was performed using the unstratified log-rank test. The HR was calculated using the unstratified Cox model with treatment as the only covariate. The CI for the hazard ratio was calculated using the profile likelihood. HR <1 favors acalabrutinib. ACAL, acalabrutinib; BICR, blinded independent central review; CI, confidence interval; CHPRI, chlorambucil plus rituximab; HR, hazard ratio; iwCLL, International Workshop on CLL; NE, not estimable; PFS, progression-free survival.

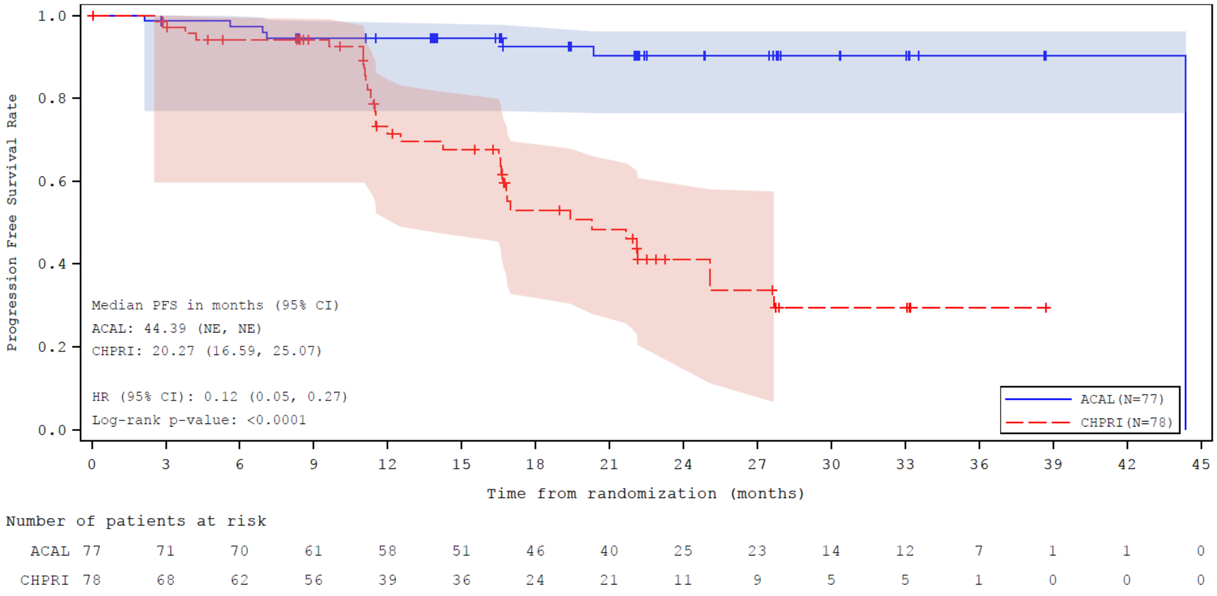

Figure S2. PFS subgroup analysis in the overall cohort. All values determining the subgroup assignments are based on baseline measurements. For the analysis in all patients, the HR was calculated using the unstratified Cox model with treatment as the only covariate. The CI for the HR was calculated using the profile likelihood. For the analysis in each subgroup, the HR was calculated using the unstratified Cox model with treatment as the only covariate. The CI for the HR was calculated using the profile likelihood. No multiplicity adjustments were made for this analysis. Acala, acalabrutinib; C+R, chlorambucil plus rituximab; CI, confidence interval; ECOG PS, Eastern Cooperative Oncology Group performance status; HR, hazard ratio; NE, not estimable; PFS, progression-free survival.

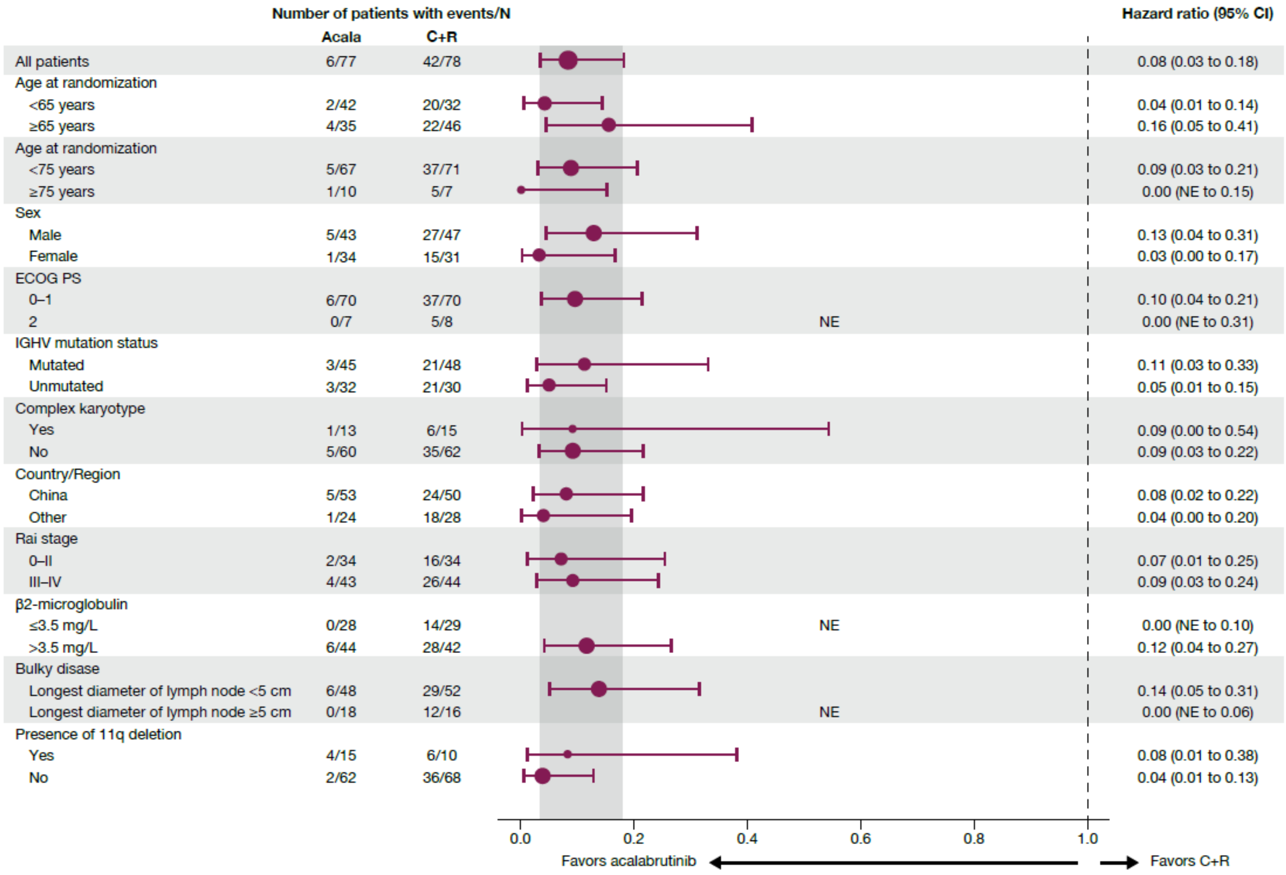

Figure S3. BICR-assessed PFS by treatment and 11q deletion in the overall cohort. Progression is determined by BICR per iwCLL 2018 criteria.[1] Median PFS was calculated using the Kaplan-Meier technique. CI for median PFS was derived based on Brookmeyer-Crowley method. ACAL, acalabrutinib; BICR, blinded independent central review; CI, confidence interval; CHPRI, chlorambucil plus rituximab; HR, hazard ratio; iwCLL, International Workshop on CLL; N, no; NE, not estimable; PFS, progression-free survival; Y, yes.

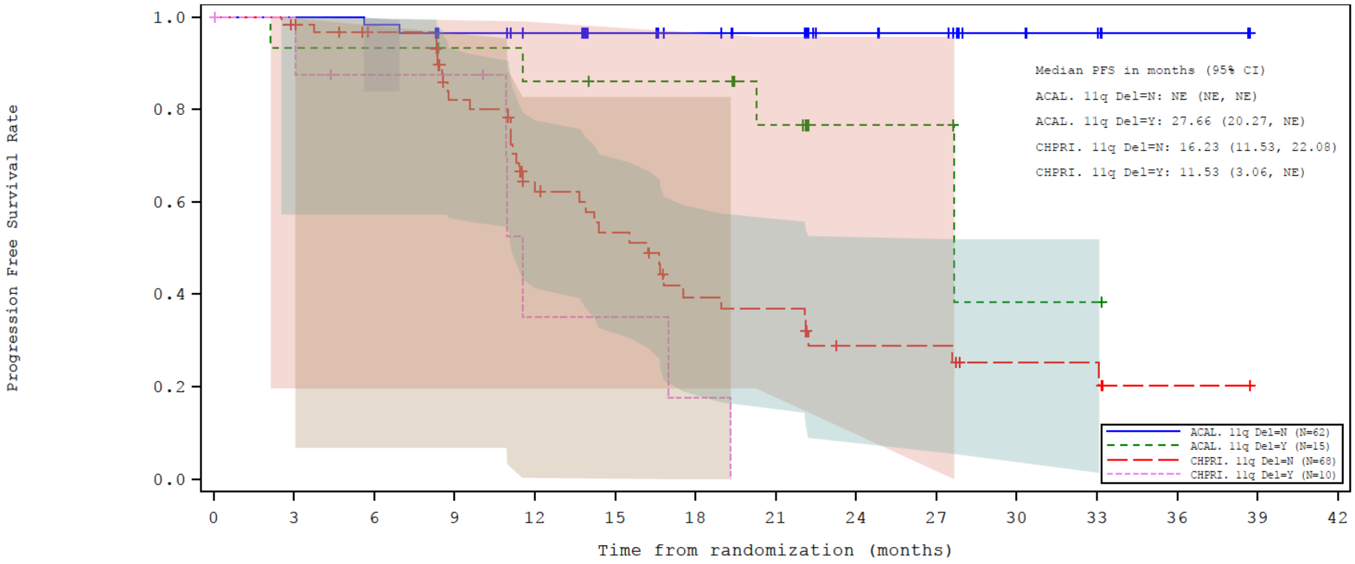

Number of patients at risk

ACAL. 11q Del=N	62	57	56	49	45	39	35	31	21	19	11	9	6	0	0
ACAL. 11q Del=Y	15	14	14	13	12	11	11	8	3	3	1	1	0	0	0
CHPRI. 11q Del=N	68	60	55	43	29	24	16	15	8	8	5	5	1	0	0
CHPRI. 11q Del=Y	10	8	6	6	2	2	1	0	0	0	0	0	0	0	0

Figure S4. BICR-assessed PFS by treatment and IGHV status in the overall cohort. Progression is determined by BICR per iwCLL 2018 criteria.[1] Median PFS was calculated using the Kaplan-Meier technique. CI for median PFS was derived based on Brookmeyer-Crowley method. ACAL, acalabrutinib; BICR, blinded independent central review; CI, confidence interval; CHPRI, chlorambucil plus rituximab; HR, hazard ratio; IGHV, immunoglobulin heavy-chain variable region genes; iwCLL, International Workshop on CLL; NE, not estimable; PFS, progression-free survival.

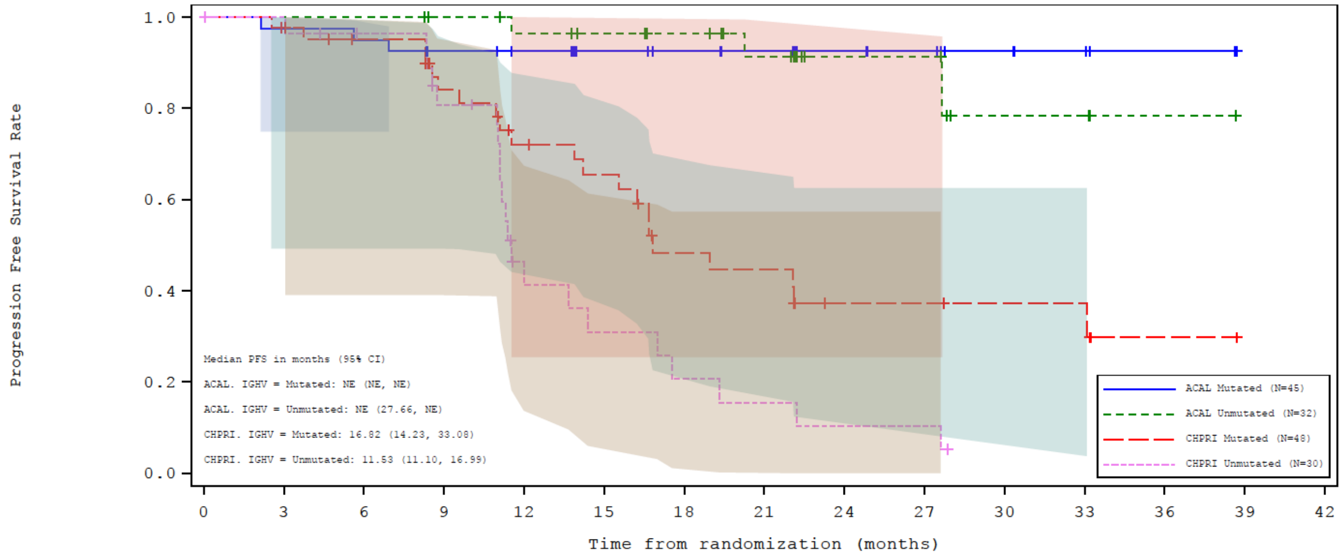

Number of patients at risk

ACAL Mutated	45	39	38	32	30	26	24	21	15	13	8	6	4	0	0
ACAL Unmutated	32	32	32	30	27	24	22	18	9	9	4	4	2	0	0
CHPRI Mutated	48	40	36	29	23	20	13	12	6	6	5	5	1	0	0
CHPRI Unmutated	30	28	25	20	8	6	4	3	2	2	0	0	0	0	0

Figure S5. BICR-assessed response rates in (A) the overall cohort and (B) the China cohort. Responses were assessed by BICR per iwCLL 2018 criteria.[1] A patient was defined to have an overall response if the best overall response was CR, CRi, nPR, or PR at or before initiation of subsequent anticancer therapy (including crossover therapy); such patients were counted in the ORR. The denominators in the response rates were based on the full analysis set. Unknown includes patients without any adequate post-baseline disease assessment. Acala, acalabrutinib; BICR, blinded independent central review; C+R, chlorambucil plus rituximab; CR, complete response; CRi, complete response with incomplete bone marrow recovery; iwCLL, International Workshop on Chronic Lymphocytic Leukemia; nPR, nodular partial response; ORR, overall response rate; PD, progressive disease; PR, partial response; PRL, partial response with lymphocytosis; SD, stable disease.

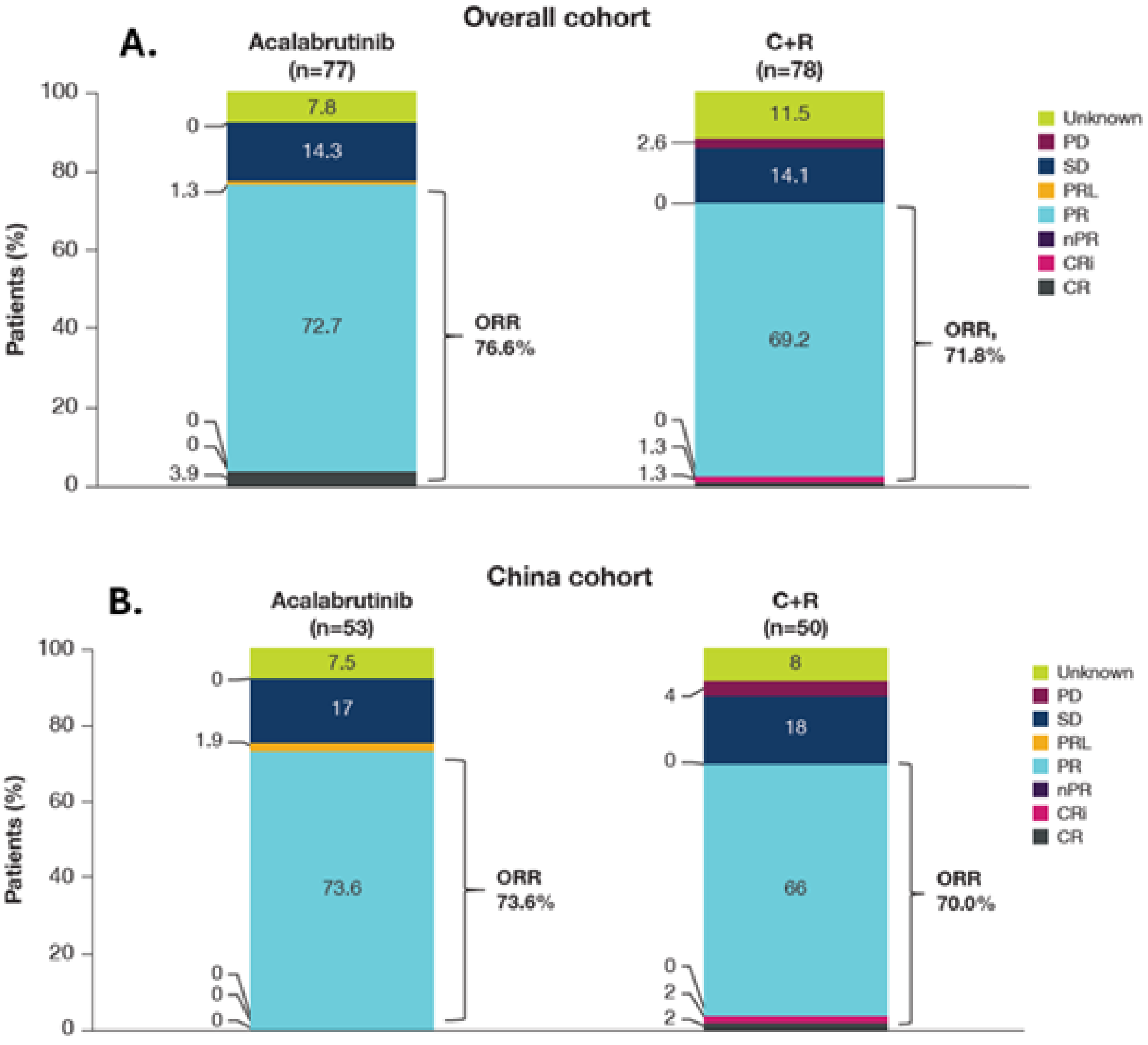

Table S1. Patient demographics and baseline characteristics.

Characteristic	Overall Cohort		China Cohort	
	**Acalabrutinib (*n*=77)**	**C+R (*n*=78)**	**Acalabrutinib (*n*=53)**	**C+R (*n*=50)**
Age, y				
Mean (SD)	62.8 (9.16)	64.1 (11.65)	62.7 (8.78)	62.6 (11.50)
Median (range)	64.0 (38–86)	67.0 (21–87)	64.0 (43–79)	65.0 (21–86)
Age group, *n* (%)				
<65	42 (54.5)	32 (41.0)	28 (52.8)	22 (44.0)
≥65 to <75	25 (32.5)	39 (50.0)	19 (35.8)	25 (50.0)
≥75	10 (13.0)	7 (9.0)	6 (11.3)	3 (6.0)
Sex, *n* (%)				
Male	43 (55.8)	47 (60.3)	30 (56.6)	32 (64.0)
Female	34 (44.2)	31 (39.7)	23 (43.4)	18 (36.0)
Race, *n* (%)				
Asian	77 (100)	75 (96.2)	53 (100)	50 (100)
White	0	3 (3.8)	0	0
ECOG PS, *n* (%)				
0–1	72 (93.5)	71 (91.0)	51 (96.2)	46 (92.0)
2	5 (6.5)	7 (9.0)	2 (3.8)	4 (8.0)
Unfit criteria, *n* (%)				
Age ≥65 y	35 (45.5)	46 (59.0)	25 (47.2)	28 (56.0)
Age >18 and <65 y	42 (54.5)	32 (41.0)	28 (52.8)	22 (44.0)
Creatinine clearance 30–69 mL/min	13 (16.9)	11 (14.1)	8 (15.1)	4 (8.0)
CIRS-G >6	32 (41.6)	27 (34.6)	23 (43.4)	20 (40.0)
Both of the above	5 (6.5)	6 (7.7)	3 (5.7)	2 (4.0)
None of the above	2 (2.6)[a]	0	0	0
Rai stage III or IV, *n* (%)	44 (57.1)	44 (56.4)	31 (58.5)	27 (54.0)
Bulky disease ≥5 cm, *n* (%)	18 (23.4)	16 (20.5)	13 (24.5)	8 (16.0)
β2-macroglobulin >3.5 mg/L, *n* (%)	44 (57.1)	42 (53.8)	30 (56.6)	25 (50.0)
Cytogenetic abnormality, *n* (%)				

del(11q)	15 (19.5)	10 (12.8)	12 (22.6)	8 (16.0)
Complex karyotype[b]	13 (16.9)	15 (19.2)	10 (18.9)	11 (22.0)
IGHV unmutated, *n* (%)	32 (41.6)	30 (38.5)	22 (41.5)	19 (38.0)
Any cytopenia, *n* (%)	42 (54.5)	51 (65.4)	30 (56.6)	30 (60.0)
ANC ≤1.5 x 10^9/L	8 (10.4)	8 (10.3)	5 (9.4)	6 (12.0)
Hemoglobin ≤11 g/dL	35 (45.5)	41 (52.6)	23 (43.4)	23 (46.0)
Platelets ≤100x10^9/L	19 (24.7)	27 (34.6)	14 (26.4)	19 (38.0)
All of the above	5 (6.5)	4 (5.1)	2 (3.8)	4 (8.0)
None of the above	35 (45.5)	27 (34.6)	23 (43.4)	20 (40.0)
Any constitutional symptoms, *n* (%)	29 (37.7)	28 (35.9)	13 (24.5)	12 (24.0)
Weight loss	12 (15.6)	9 (11.5)	4 (7.5)	2 (4.0)
Fever	2 (2.6)	0	0	0
Night sweats	16 (20.8)	18 (23.1)	8 (15.1)	7 (14.0)
Fatigue	10 (13.0)	10 (12.8)	3 (5.7)	5 (10.0)
None of the above	48 (62.3)	50 (64.1)	40 (75.5)	38 (76.0)

[a]Two patients in the overall acalabrutinib arm had creatinine clearance <69 mL/min during the screening period, which met the inclusion criteria; their creatinine clearances improved spontaneously and transiently on cycle 1 day 1.

[b]Defined as patients with 3 or more abnormalities with ≥1 structural abnormality excluding inversion of chromosome 9.

ANC, absolute neutrophil count; C+R, chlorambucil + rituximab; CIRS-G, Cumulative Illness Rating Scale–Geriatric; ECOG PS, Eastern Cooperative Oncology Group performance status; IGHV, immunoglobulin heavy-chain variable region genes; SD, standard deviation.

Table S2. Summary of COVID-19 study disruptions (ITT population).

	**Overall Cohort**		
***n* (%)**	**Acalabrutinib (*n*=77)**	**C+R (*n*=78)**	**Total (*N*=155)**
Patients with ≥1 disruption due to COVID-19 pandemic	27 (35.1)	17 (21.8)	44 (28.4)
Patients with visit impacted	26 (33.8)	17 (21.8)	43 (27.7)
Patients with study drug impacted	9 (11.7)	4 (5.1)	13 (8.4)
Patients who withdrew from study due to COVID-19 pandemic	0	0	0

C+R, chlorambucil plus rituximab; ITT, intention-to-treat.

Table S3. Best overall response and duration of response, assessed by investigator (ITT population).

	**Overall Cohort**		**China Cohort**	
	**Acalabrutinib (*n*=77)**	**C+R (*n*=78)**	**Acalabrutinib (*n*=53)**	**C+R (*n*=50)**
ORR, *n* (%)	63 (81.8)	47 (60.3)	42 (79.2)	28 (56.0)
Odds ratio (95% CI)	2.94 (1.41, 6.11)		2.67 (1.13, 6.33)	
2-sided *P*-value	0.0034		0.0246	
Best overall response, *n* (%)				
CR	7 (9.1)	1 (1.3)	1 (1.9)	1 (2.0)
CRi	0	0	0	0
nPR	0	0	0	0
PR	56 (72.7)	46 (59.0)	41 (77.4)	27 (54.0)
PRL	2 (2.6)	0	2 (3.8)	0
SD	8 (10.4)	20 (25.6)	7 (13.2)	16 (32.0)
PD	0	2 (2.6)	0	2 (4.0)
Unknown	4 (5.2)	9 (11.5)	2 (3.8)	4 (8.0)
Responders who subsequently progressed or died, *n* (%)				
Progression	5 (7.9)	22 (46.8)	5 (11.9)	13 (46.4)
Death without progression	0	0	0	0
Median duration of response, months (95% CI)	NR (30.55, NE)	19.29 (11.47, NE)	30.55 (NE, NE)	14.52 (8.61, NE)
Hazard ratio (95% CI)	0.15 (0.05, 0.36)		0.17 (0.05, 0.49)	
Log-rank *P*-value	<0.0001		0.0005	

Unknown includes patients without any adequate post-baseline disease assessment.
Responses are assessed by investigator per iwCLL 2018 criteria.[1]
Hazard ratio <1 for duration of response favors acalabrutinib.
C+R, chlorambucil plus rituximab; CI, confidence interval; CR, complete response; CRi, complete response with incomplete bone marrow recovery; ITT, intention-to-treat; iwCLL, International Workshop on CLL; NE, not evaluable; nPR nodular partial response; NR, not reached; PD, progressive disease; PR, partial response; PRL, partial response with lymphocytosis; SD stable disease.

Table S4. AEs leading to dose interruption or reduction of any treatment by system organ class and preferred term (safety population).

	Overall Cohort		China Cohort	
***n* (%)**	**Acalabrutinib (*n*=77)**	**C+R (*n*=73)**	**Acalabrutinib (*n*=53)**	**C+R (*n*=47)**
**Dose interruptions**				
Patients with any AE leading to dose interruption	32 (41.6)	18 (24.7)	20 (37.7)	13 (27.7)
Infections and infestations	20 (26.0)	3 (4.1)	11 (20.8)	1 (2.1)
Central nervous system infection	1 (1.3)	0	1 (1.9)	0
COVID-19	13 (16.9)	1 (1.4)	9 (17.0)	0
COVID-19 pneumonia	3 (3.9)	0	2 (3.8)	0
Dacryocystitis	1 (1.3)	0	1 (1.9)	0
Fournier's gangrene	1 (1.3)	0	0	0
Fungal infection	1 (1.3)	0	0	0
Hepatitis B reactivation	3 (3.9)	1 (1.4)	0	1 (2.1)
Pneumonia	6 (7.8)	1 (1.4)	4 (7.5)	0
Wound infection	1 (1.3)	0	0	0
Blood and lymphatic system disorders	4 (5.2)	4 (5.5)	1 (1.9)	3 (6.4)
Anemia	3 (3.9)	1 (1.4)	1 (1.9)	1 (2.1)
Febrile neutropenia	0	1 (1.4)	0	1 (2.1)
Neutropenia	2 (2.6)	3 (4.1)	0	2 (4.3)
Thrombocytopenia	3 (3.9)	0	0	0
Immune system disorders	0	1 (1.4)	0	0
Hypersensitivity	0	1 (1.4)	0	0
Metabolism and nutrition disorders	1 (1.3)	1 (1.4)	1 (1.9)	1 (2.1)
Diabetes mellitus	0	1 (1.4)	0	1 (2.1)
Hyperkalemia	1 (1.3)	0	1 (1.9)	0
Hyponatremia	1 (1.3)	0	1 (1.9)	0
Nervous system disorders	3 (3.9)	0	3 (5.7)	0

Carotid artery stenosis	1 (1.3)	0	1 (1.9)	0
Headache	1 (1.3)	0	1 (1.9)	0
Intracranial aneurysm	1 (1.3)	0	1 (1.9)	0
Eye disorders	1 (1.3)	0	1 (1.9)	0
Dacryoadenitis acquired	1 (1.3)	0	1 (1.9)	0
Eye swelling	1 (1.3)	0	1 (1.9)	0
Ear and labyrinth disorders	1 (1.3)	0	0	0
Vertigo positional	1 (1.3)	0	0	0
Cardiac disorders	1 (1.3)	0	0	0
Coronary artery disease	1 (1.3)	0	0	0
Hepatobiliary disorders	1 (1.3)	0	1 (1.9)	0
Hepatic function abnormal	1 (1.3)	0	1 (1.9)	0
Skin and subcutaneous tissue disorders	5 (6.5)	2 (2.7)	5 (9.4)	2 (4.3)
Dermatitis allergic	2 (2.6)	1 (1.4)	2 (3.8)	1 (2.1)
Drug eruption	1 (1.3)	0	1 (1.9)	0
Hemorrhage subcutaneous	1 (1.3)	0	1 (1.9)	0
Pruritus	1 (1.3)	1 (1.4)	1 (1.9)	1 (2.1)
Musculoskeletal and connective tissue disorders	1 (1.3)	0	0	0
Musculoskeletal chest pain	1 (1.3)	0	0	0
Renal and urinary disorders	1 (1.3)	0	1 (1.9)	0
Acute kidney injury	1 (1.3)	0	1 (1.9)	0
General disorders and administration site conditions	1 (1.3)	1 (1.4)	0	0
Chills	0	1 (1.4)	0	0
Pyrexia	1 (1.3)	0	0	0
Investigations	5 (6.5)	6 (8.2)	3 (5.7)	5 (10.6)

Alanine aminotransferase increased	1 (1.3)	0	1 (1.9)	0
Hepatic enzyme increased	2 (2.6)	0	0	0
Neutrophil count decreased	2 (2.6)	6 (8.2)	2 (3.8)	5 (10.6)
Platelet count decreased	1 (1.3)	1 (1.4)	1 (1.9)	1 (2.1)
White blood cell count decreased	0	1 (1.4)	0	1 (2.1)
Injury, poisoning and procedural complications	2 (2.6)	4 (5.5)	0	3 (6.4)
Infusion-related reaction	0	4 (5.5)	0	3 (6.4)
Ligament sprain	1 (1.3)	0	0	0
Traumatic intracranial hemorrhage	1 (1.3)	0	0	0
**Dose reductions**				
Patients with any AE leading to dose reduction	0	9 (12.3)	0	5 (10.6)
Blood and lymphatic system disorders	0	4 (5.5)	0	1 (2.1)
Anemia	0	1 (1.4)	0	0
Neutropenia	0	2 (2.7)	0	1 (2.1)
Thrombocytopenia	0	1 (1.4)	0	0
Investigations	0	5 (6.8)	0	4 (8.5)
Neutrophil count decreased	0	4 (5.5)	0	3 (6.4)
Platelet count decreased	0	2 (2.7)	0	2 (4.3)
White blood cell count decreased	0	1 (1.4)	0	1 (2.1)

Includes AEs with an onset date, or pre-treatment AEs that increase in severity, on or after the date of first dose and up to and including 30 days following the date of last dose of study treatment or up to the date of initiation of the first subsequent anticancer therapy (including crossover treatment), whichever occurs first.
AE, adverse event; C+R, chlorambucil plus rituximab.

Table S5. AEs leading to treatment discontinuation by system organ class and preferred term (safety population).

	Overall Cohort		China Cohort	
***n* (%)**	**Acalabrutinib (*n*=77)**	**C+R (*n*=73)**	**Acalabrutinib (*n*=53)**	**C+R (*n*=47)**
Patients with any AE leading to discontinuation	8 (10.4)	8 (11.0)	6 (11.3)	3 (6.4)
Infections and infestations	4 (5.2)	2 (2.7)	3 (5.7)	1 (2.1)
COVID-19 pneumonia	0	1 (1.4)	0	0
Hepatitis B reactivation	3 (3.9)	1 (1.4)	2 (3.8)	1 (2.1)
Pneumonia	1 (1.3)	0	1 (1.9)	0
Neoplasms benign, malignant and unspecified (including cysts and polyps)	1 (1.3)	0	1 (1.9)	0
Breast cancer	1 (1.3)	0	1 (1.9)	0
Blood and lymphatic system disorders	0	4 (5.5)	0	2 (4.3)
Anemia	0	2 (2.7)	0	2 (4.3)
Neutropenia	0	2 (2.7)	0	0
Metabolism and nutrition disorders	0	1 (1.4)	0	0
Tumor lysis syndrome	0	1 (1.4)	0	0
Skin and subcutaneous tissue disorders	2 (2.6)	0	1 (1.9)	0
Purpura	1 (1.3)	0	1 (1.9)	0
Rash maculopapular	1 (1.3)	0	0	0
Renal and urinary disorders	0	1 (1.4)	0	0
Acute kidney injury	0	1 (1.4)	0	0
Investigations	1 (1.3)	0	1 (1.9)	0
Platelet count decreased	1 (1.3)	0	1 (1.9)	0

Patients with multiple AEs leading to study drug discontinuation are counted once for each system organ class.

Includes AEs with an onset date, or pretreatment AEs that increase in severity, on or after the date of first dose and up to and including 30 days following the date of last dose of study treatment or up to the date of initiation of the first subsequent anticancer therapy (including crossover treatment), whichever occurs

first. Events that led to discontinuation of either chlorambucil or rituximab are considered in the calculation for the C+R arm.
AE, adverse event; C+R, chlorambucil+rituximab.

Table S6. Serious AEs by system organ class and preferred term (n >1 in any treatment arm) (safety population).

	**Overall Cohort**		**China Cohort**	
***n* (%)**	**Acalabrutinib (*n*=77)**	**C+R (*n*=73)**	**Acalabrutinib (*n*=53)**	**C+R (*n*=47)**
Patients with any SAE	30 (39.0)	17 (23.3)	18 (34.0)	10 (21.3)
Infections and infestations	21 (27.3)	6 (8.2)	13 (24.5)	4 (8.5)
Bronchitis	0	1 (1.4)	0	1 (2.1)
Cellulitis	1 (1.3)	0	1 (1.9)	0
Central nervous system infection	1 (1.3)	0	1 (1.9)	0
COVID-19	6 (7.8)	0	3 (5.7)	0
COVID-19 pneumonia	5 (6.5)	1 (1.4)	4 (7.5)	0
Fournier's gangrene	1 (1.3)	0	0	0
Pneumonia	7 (9.1)	4 (5.5)	5 (9.4)	3 (6.4)
Salmonellosis	1 (1.3)	0	1 (1.9)	0
Sepsis	1 (1.3)	0	1 (1.9)	0
Septic shock	0	1 (1.4)	0	0
Wound infection	1 (1.3)	0	0	0
Neoplasms benign, malignant and unspecified (incl cysts and polyps)	1 (1.3)	0	1 (1.9)	0
Breast cancer	1 (1.3)	0	1 (1.9)	0
Blood and lymphatic system disorders	4 (5.2)	4 (5.5)	1 (1.9)	2 (4.3)
Anemia	3 (3.9)	2 (2.7)	1 (1.9)	1 (2.1)
Febrile neutropenia	1 (1.3)	3 (4.1)	0	2 (4.3)
Leukocytosis	1 (1.3)	0	0	0
Neutropenia	2 (2.6)	1 (1.4)	0	0
Thrombocytopenia	2 (2.6)	0	0	0
Immune system disorders	0	1 (1.4)	0	0
Hypersensitivity	0	1 (1.4)	0	0

Metabolism and nutrition disorders	0	2 (2.7)	0	1 (2.1)
Tumor lysis syndrome	0	2 (2.7)	0	1 (2.1)
Nervous system disorders	3 (3.9)	1 (1.4)	2 (3.8)	1 (2.1)
Carotid artery stenosis	1 (1.3)	0	1 (1.9)	0
Dizziness	1 (1.3)	1 (1.4)	0	1 (2.1)
Intracranial aneurysm	1 (1.3)	0	1 (1.9)	0
Cardiac disorders	2 (2.6)	0	0	0
Coronary artery disease	1 (1.3)	0	0	0
Supraventricular extrasystoles	1 (1.3)	0	0	0
Ventricular extrasystoles	1 (1.3)	0	0	0
Respiratory, thoracic and mediastinal disorders	2 (2.6)	1 (1.4)	1 (1.9)	0
Bronchostenosis	1 (1.3)	0	0	0
Chronic obstructive pulmonary disease	0	1 (1.4)	0	0
Pleural effusion	1 (1.3)	0	1 (1.9)	0
Hepatobiliary disorders	2 (2.6)	1 (1.4)	2 (3.8)	0
Bile duct stone	1 (1.3)	0	1 (1.9)	0
Hepatic function abnormal	1 (1.3)	0	1 (1.9)	0
Liver injury	0	1 (1.4)	0	0
Skin and subcutaneous tissue disorders	2 (2.6)	0	1 (1.9)	0
Drug eruption	1 (1.3)	0	1 (1.9)	0
Rash maculopapular	1 (1.3)	0	0	0
Musculoskeletal and connective tissue disorders	1 (1.3)	0	1 (1.9)	0
Intervertebral disc protrusion	1 (1.3)	0	1 (1.9)	0
Renal and urinary disorders	1 (1.3)	1 (1.4)	1 (1.9)	0

Acute kidney injury	1 (1.3)	1 (1.4)	1 (1.9)	0
General disorders and administration site conditions	1 (1.3)	1 (1.4)	0	1 (2.1)
Pyrexia	1 (1.3)	1 (1.4)	0	1 (2.1)
Investigations	3 (3.9)	3 (4.1)	1 (1.9)	2 (4.3)
Hepatic enzyme increased	2 (2.6)	0	0	0
Neutrophil count decreased	0	2 (2.7)	0	1 (2.1)
Platelet count decreased	1 (1.3)	2 (2.7)	1 (1.9)	2 (4.3)
White blood cell count decreased	0	1 (1.4)	0	1 (2.1)
Injury, poisoning and procedural complications	1 (1.3)	2 (2.7)	0	1 (2.1)
Infusion related reaction	0	1 (1.4)	0	0
Ligament sprain	1 (1.3)	0	0	0
Wrist fracture	0	1 (1.4)	0	1 (2.1)

Patients with multiple SAEs are counted once for each system organ class/preferred term.
Includes AEs with an onset date, or pretreatment AEs that increase in severity, on or after the date of first dose and up to and including 30 days following the date of last dose of study treatment or up to the date of initiation of the first subsequent anticancer therapy (including crossover treatment), whichever occurs first.
AE, adverse event; C+R, chlorambucil plus rituximab; SAE, serious adverse event.

Table S7. Exposure-adjusted AEs in any category (safety population).

AE category, *n* (%)	AEs per 100 patient-years	
	**Acalabrutinib (*n*=77)**	**C+R (*n*=73)**
Any AE	689.33	892.13
Any causally related AEs	137.13	346.89
Any AE leading to death	0.92	2.77
Any causally related AE leading to death	0	0
Any AE of grade 3 or higher	69.07	96.14
Any causally related AE of grade 3 or higher	40.09	67.53
Any SAE	41.21	52.87
Any causally related SAE	13.36	36.8
Any AE leading to discontinuation of study drug	7.44	22.99
Any causally related AE leading to discontinuation of study drug	3.68	17.21
Any AE leading to dose interruption	46.15	59.43
Any causally related AE leading to dose interruption	18.41	47.45
Any AE leading to dose reduction	0	26.85
Any causally related AE leading to dose reduction	0	26.85

AE, adverse event; C+R, chlorambucil plus rituximab; SAE, serious adverse event.

Table S8. Overall summary of events of clinical interest (safety population).

	Overall Cohort				China Cohort			
***n* (%)**	**Acalabrutinib (*n*=77)**		**C+R (*n*=73)**		**Acalabrutinib (*n*=53)**		**C+R (*n*=47)**	
	**Any Grade**	**Grade ≥3**	**Any Grade**	**Grade ≥3**	**Any Grade**	**Grade ≥3**	**Any Grade**	**Grade ≥3**
Patients with any event of clinical interest	60 (77.9)	30 (39.0)	51 (69.9)	24 (32.9)	39 (73.6)	19 (35.8)	34 (72.3)	17 (36.2)
Cardiac events	7 (9.1)	2 (2.6)	2 (2.7)	0	4 (7.5)	1 (1.9)	0	0
Atrial fibrillation	0	0	0	0	0	0	0	0
Ventricular tachyarrhythmias	3 (3.9)	1 (1.3)	0	0	2 (3.8)	1 (1.9)	0	0
Cytopenia (anemia)	12 (15.6)	7 (9.1)	12 (16.4)	6 (8.2)	7 (13.2)	4 (7.5)	9 (19.1)	5 (10.6)
Cytopenia (leukopenia)	16 (20.8)	8 (10.4)	32 (43.8)	17 (23.3)	12 (22.6)	5 (9.4)	22 (46.8)	12 (25.5)
Neutropenia	14 (18.2)	8 (10.4)	30 (41.1)	17 (23.3)	10 (18.9)	5 (9.4)	20 (42.6)	12 (25.5)
Other leukopenia	4 (5.2)	1 (1.3)	10 (13.7)	4 (5.5)	3 (5.7)	0	10 (21.3)	4 (8.5)
Cytopenia (thrombocytopenia)	15 (19.5)	7 (9.1)	12 (16.4)	4 (5.5)	11 (20.8)	5 (9.4)	9 (19.1)	4 (8.5)
Hemorrhage	16 (20.8)	1 (1.3)	4 (5.5)	0	12 (22.6)	1 (1.9)	3 (6.4)	0
Major hemorrhage	2 (2.6)	1 (1.3)	0	0	1 (1.9)	1 (1.9)	0	0
Hypertension	0	0	1 (1.4)	0	0	0	1 (2.1)	0
Infections	52 (67.5)	17 (22.1)	27 (37.0)	5 (6.8)	34 (64.2)	10 (18.9)	20 (42.6)	4 (8.5)
Interstitial lung disease/pneumonitis	1 (1.3)	0	0	0	1 (1.9)	0	0	0
Secondary primary malignancies	1 (1.3)	0	0	0	1 (1.9)	0	0	0
Secondary primary malignancies (excluding non-melanoma skin)	1 (1.3)	0	0	0	1 (1.9)	0	0	0

Tumor lysis syndrome	0	0	2 (2.7)	2 (2.7)	0	0	1 (2.1)	1 (2.1)

Patients with multiple ECIs are counted once for each category/subcategory.
Includes AEs with an onset date, or pretreatment AEs that increase in severity, on or after the date of first dose and up to and including 30 days following the date of last dose of study treatment or up to the date of initiation of the first subsequent anticancer therapy (including crossover treatment), whichever occurs first.
AE, adverse event; C+R, chlorambucil + rituximab; ECI, event of clinical interest.

Table S9. Cardiac events (any grade and grade ≥3) by preferred term (safety population).

	**Overall Cohort**				**China Cohort**			
***n* (%)**	**Acalabrutinib (*n*=77)**		**C+R (*n*=73)**		**Acalabrutinib (*n*=53)**		**C+R (*n*=47)**	
	**Any Grade**	**Grade ≥3**	**Any Grade**	**Grade ≥3**	**Any Grade**	**Grade ≥3**	**Any Grade**	**Grade ≥3**
Cardiac events	7 (9.1)	2 (2.6)	2 (2.7)	0	4 (7.5)	1 (1.9)	0	0
Sinus tachycardia	0	0	2 (2.7)	0	0	0	0	0
Supraventricular extrasystoles	2 (2.6)	0	0	0	1 (1.9)	0	0	0
Bundle branch block left	1 (1.3)	0	0	0	1 (1.9)	0	0	0
Bundle branch block right	1 (1.3)	0	0	0	0	0	0	0
Coronary artery disease	1 (1.3)	1 (1.3)	0	0	0	0	0	0
Myocardial ischaemia	1 (1.3)	0	0	0	0	0	0	0
Palpitations	1 (1.3)	0	0	0	1 (1.9)	0	0	0
Ventricular tachyarrhythmias	3 (3.9)	1 (1.3)	0	0	2 (3.8)	1 (1.9)	0	0
Ventricular extrasystoles	2 (2.6)	0	0	0	1 (1.9)	0	0	0
Ventricular arrhythmia	1 (1.3)	1 (1.3)	0	0	1 (1.9)	1 (1.9)	0	0
Ventricular tachycardia	1 (1.3)	1 (1.3)	0	0	1 (1.9)	1 (1.9)	0	0

Patients with multiple ECIs are counted once for each category/subcategory.

Includes AEs with an onset date, or pretreatment AEs that increase in severity, on or after the date of first dose and up to and including 30 days following the date of last dose of study treatment or up to the date of initiation of the first subsequent anticancer therapy (including crossover treatment), whichever occurs first.

AE, adverse event; C+R, chlorambucil+rituximab; ECI, event of clinical interest.

Table S10. Grade ≥3 infections by preferred term (n >1 event in combined treatment arms) (safety population).

	Overall Cohort		China Cohort	
n **(%)**	**Acalabrutinib (*n*=77)**	**C+R (*n*=73)**	**Acalabrutinib (*n*=53)**	**C+R (*n*=47)**
Any grade ≥3 infections	17 (22.1)	5 (6.8)	10 (18.9)	4 (8.5)
Pneumonia	6 (7.8)	4 (5.5)	5 (9.4)	4 (8.5)
COVID-19 pneumonia	5 (6.5)	0	4 (7.5)	0
COVID 19	4 (5.2)	0	1 (1.9)	0
Cellulitis	1 (1.3)	0	1 (1.9)	0
Central nervous system infection	1 (1.3)	0	1 (1.9)	0
Fournier's gangrene	1 (1.3)	0	0	0
Sepsis	1 (1.3)	0	1 (1.9)	0
Septic shock	0	1 (1.4)	0	0
Upper respiratory tract infection	1 (1.3)	0	1 (1.9)	0
Wound infection	1 (1.3)	0	0	0

Patients with multiple ECIs are counted once for each category/subcategory. Includes AEs with an onset date, or pretreatment AEs that increase in severity, on or after the date of first dose and up to and including 30 days following the date of last dose of study treatment or up to the date of initiation of the first subsequent anticancer therapy (including crossover treatment), whichever occurs first.
AE, adverse event; C+R, chlorambucil+rituximab; ECI, event of clinical interest.

Table S11. Deaths (ITT population).

	Overall Cohort		China Cohort	
n (%)	Acalabrutinib (*n*=77)	C+R (*n*=73)	Acalabrutinib (*n*=53)	C+R (*n*=47)
Death	2 (2.6)	4 (5.5)	1 (1.9)	1 (2.1)
Primary cause of death				
AE	1 (1.3)	2 (2.7)	1 (1.9)	0
Related to disease under investigation[a]	0	1 (1.4)	0	1 (2.1)
Other	1 (1.3)	1 (1.4)	0	0
Death within 30 days of last dose	1 (1.3)	0	1 (1.9)	0
Primary cause of death				
AE	1 (1.3)	0	1 (1.9)	0
Sepsis	1 (1.3)	0	1 (1.9)	0
Death more than 30 days of last dose	1 (1.3)	4 (5.5)	0	1 (2.1)
Primary cause of death				
AE	0	2 (2.7)	0	0
Septic Shock	0	1 (1.4)	0	0
Pneumonia	0	1 (1.4)	0	0
Related to disease under investigation[a]	0	1 (1.4)	0	1 (2.1)
Sepsis	0	1 (1.4)	0	1 (2.1)
Other	1 (1.3)	1 (1.4)	0	0
Mantle cell lymphoma progression	1 (1.3)	0	0	0
Hospital-acquired pneumonia	0	1 (1.4)	0	0

[a]Death related to disease under investigation is determined by the investigator.
Rows are mutually exclusive; patients are only reported in one category.
AE, adverse event; C+R, chlorambucil plus rituximab; ITT, intention-to-treat.

Reference

1. Hallek M, Cheson BD, Catovsky D, Caligaris-Cappio F, Dighiero G, Döhner H, Hillmen P, Keating M, Montserrat E, Chiorazzi N, Stilgenbauer S, Rai KR, Byrd JC, Eichhorst B, O'Brien S, Robak T, Seymour JF, Kipps TJ (2018) iwCLL guidelines for diagnosis, indications for treatment, response assessment, and supportive management of CLL. Blood 131(25):2745-2760. 10.1182/blood-2017-09-806398
